# A single-cell atlas of liver metastases of colorectal cancer reveals reprogramming of the tumor microenvironment in response to preoperative chemotherapy

**DOI:** 10.1038/s41421-021-00312-y

**Published:** 2021-09-07

**Authors:** Li-Heng Che, Jing-Wen Liu, Jian-Ping Huo, Rong Luo, Rui-Ming Xu, Cai He, Yu-Qing Li, Ai-Jun Zhou, Piao Huang, Yong-Yu Chen, Wen Ni, Yun-Xia Zhou, Yuan-Yuan Liu, Hui-Yan Li, Rong Zhou, Hui Mo, Jian-Ming Li

**Affiliations:** 1 Department of Pathology, Sun Yat-Sen Memorial Hospital, Sun Yat-Sen University, Guangzhou, Guangdong China; 2 Guangdong Provincial Key Laboratory of Malignant Tumor Epigenetics and Gene Regulation, Sun Yat-Sen Memorial Hospital, Sun Yat-Sen University, Guangzhou, Guangdong China

**Keywords:** Cancer microenvironment, Colorectal cancer, Chemotherapy

## Abstract

Metastasis is the primary cause of cancer-related mortality in colorectal cancer (CRC) patients. How to improve therapeutic options for patients with metastatic CRC is the core question for CRC treatment. However, the complexity and diversity of stromal context of the tumor microenvironment (TME) in liver metastases of CRC have not been fully understood, and the influence of stromal cells on response to chemotherapy is unclear. Here we performed an in-depth analysis of the transcriptional landscape of primary CRC, matched liver metastases and blood at single-cell resolution, and a systematic examination of transcriptional changes and phenotypic alterations of the TME in response to preoperative chemotherapy (PC). Based on 111,292 single-cell transcriptomes, our study reveals that TME of treatment-naïve tumors is characterized by the higher abundance of less-activated B cells and higher heterogeneity of tumor-associated macrophages (TAMs). By contrast, in tumors treated with PC, we found activation of B cells, lower diversity of TAMs with immature and less activated phenotype, lower abundance of both dysfunctional T cells and ECM-remodeling cancer-associated fibroblasts, and an accumulation of myofibroblasts. Our study provides a foundation for future investigation of the cellular mechanisms underlying liver metastasis of CRC and its response to PC, and opens up new possibilities for the development of therapeutic strategies for CRC.

## Introduction

Metastasis is the primary cause of cancer-related mortality in colorectal cancer (CRC) patients^1,2^. The 5-year survival rate of CRC patients at the advanced stage (stage IV) is only about 12%^3^. In CRC, liver is the most frequent site of metastases. For the patients of metastases of colorectal cancer (mCRC), surgical resection of both primary and metastases is the best option for curative treatment^4–8^. However, mainly for the size, number, and location of liver metastases, only a minority of patients is suitable for upfront surgery (~20%)^9,10^. Moreover, even after resection, due to the latent disseminated tumor cells after surgery, relapse is very common (occurs in 75% of patients)^2,11,12^. Thus, surgery in combination with chemotherapy and/or immunotherapy become an accepted standard of care for CRC patients with liver metastases.

Preoperative chemotherapy (PC) aims at reducing tumor load, which may reduce the risk of local relapse and converting patients with initially unresectable mCRC to resectable liver metastases^13,14^. Nevertheless, despite theoretical benefit and randomized trail demonstrations^15^, whether the patients undergoing chemotherapy and resection have long-term benefit is still questionable. How to provide optimal treatment for CRC patients with liver metastasis remains a pivotal issue.

Understanding the complex cellular and phenotypic diversity within the tumor microenvironment (TME) may pave the way for the development of effective treatment for cancer, especially in metastatic disease. Recently, single-cell RNA sequencing greatly contributes to our understanding of TME in many cancers, including melanoma^16–18^, head and neck cancer^19^, hepatocellular carcinoma^20,21^, lung carcinoma^22–24^, breast carcinoma^25–28^, kidney cancer^29^, and basal cell carcinoma^30^. In CRC, single-cell genomic^31,32^, transcriptomic^33–36^, and epigenomic analyses^36^ have provided insights into intra-tumor genomic diversity and inter-tumor difference. Despite recent advances in our understanding of CRC, the cellular milieu of liver metastases and their primary counterparts are still poorly understood. How TME responds to chemotherapy in primary tumor and their corresponding liver metastases is largely unexplored.

In this study, we established a landscape of TME of liver metastases of CRC based on 111,292 single cells, and uncovered the transcriptional changes and phenotypic alteration of TME in response to chemotherapy. We found that PC may promote the activation of B cells, drive down the diversity of tumor-associated macrophages (TAMs), recruit more immature TAMs, MHC^low^ TAMs and myofibroblast, and decrease the abundance of dysfunctional T cells and ECM-remodeling cancer-associated fibroblasts (CAFs). We also find the key ligand-receptor (LR)-based cellular interactions in the cellular milieu of tumors treated with PC and treatment-naïve tumors, in both primary and the metastases of CRC. Taken together, we established a single-cell atlas of TME in both primary CRC and matched liver metastases with or without chemotherapy. This resource provides a foundation to investigate the cellular mechanisms of liver metastasis and therapeutic response, and facilitate the development of novel treatment for mCRC.

## Results

### Single-cell analysis of TME of liver metastases of colorectal cancer

To gain a better understanding of TME, and investigate how TME responds to PC in liver metastases of CRC, we performed scRNA-seq of 15 samples from three sites (primary CRC, matched liver metastases, and blood) of six CRC patients with liver metastases (Supplementary Table S1). While patients COL15, COL17, and COL18 had been treated with PC, the others were treatment naïve. All patients were classified as microsatellite-stable (MSS) with invasive adenocarcinomas and late-stage (IV) disease. Detailed information is available in Materials and Methods and Supplementary Table S1.

**Fig. 1:**
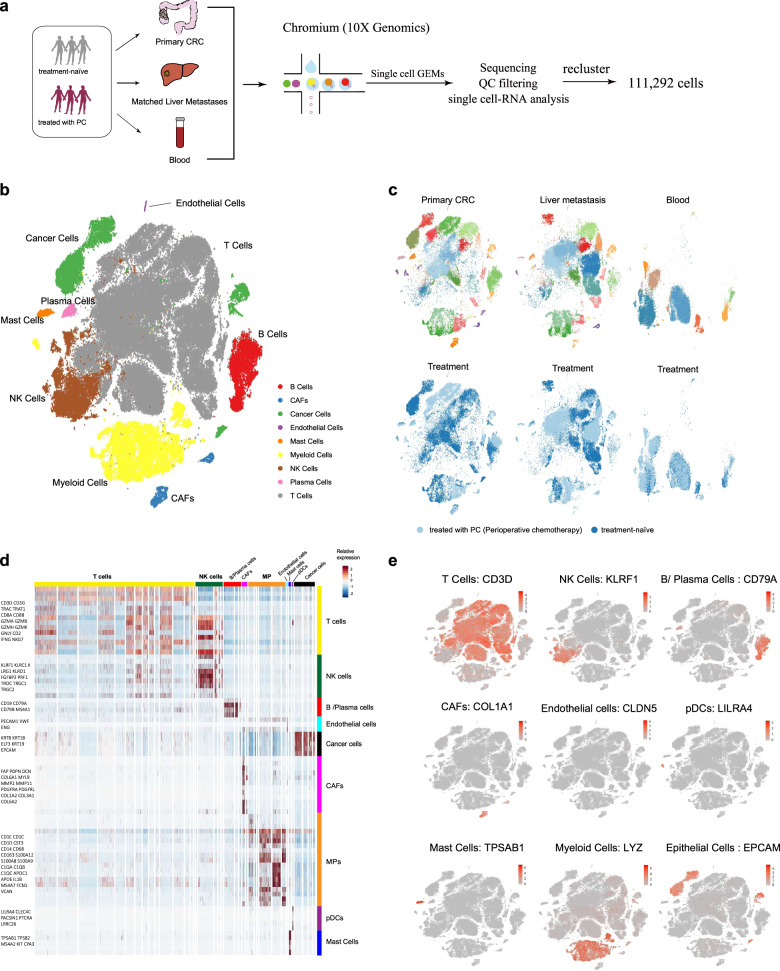
Single-cell atlas of primary CRC and liver metastases. **a** Overview of the workflow for single-cell transcriptome profiling of cells in primary CRC, matched liver metastases, and blood. **b** t-SNE plot showing the transcriptome landscape of 111,292 stromal cells from six CRC patients with liver metastasis. Colors indicate cell types. **c** t-SNE plot showing stromal cells colored according to tissue origins (top) and treatment status (bottom). **d** Heat map showing differential expression of the top expressed genes in each cluster. For each cluster, the top 10 genes and their relative expression are shown. MPs, mononuclear phagocytes; pDCs, plasmacytoid dendritic cells. **e** t-SNE plots showing typical markers of each major cell type.

Viable single cells were sorted and used for droplet-based scRNA-seq. After quality control (see Materials and Methods section), we obtained transcriptome data for 111,292 single cells from primary CRC (*n* = 6), matched liver metastases (*n* = 6), and peripheral blood mononuclear cells (PBMCs) (*n* = 3) (Supplementary Table S2). Then we clustered single cells using shared nearest neighbor clustering based on significant principal components, and visualized cell clusters using t-distributed stochastic neighbor embedding (t-SNE) (Fig. 1a; Materials and Methods section). The major cell populations (including T cells/natural killer (NK) cells, B/plasma cells, CAFs, endothelial, and myeloid cells) were annotated with canonical marker genes (Fig. 1b). Treatment state and tissue origin are mapped in Fig. 1c (also see Supplementary Fig. S1a); selected representative markers of each cell type are presented in Fig. 1d, e and Supplementary Fig. S1b.

To characterize the ecosystems of primary and metastatic CRC tumors, and better understand how TME responds to PC in liver metastases of CRC, we focused on major cell types of TME (T/NK cells, B cells, Myeloid cells, CAFs, and epithelial cells (EPCs)). For each compartment, we re-centered, scaled, normalized, and re-clustered the data. Ultimately, we obtained 28 myeloid clusters (6 dendritic cells, 18 TAMs, 1 monocytes, 2 myeloid-derived suppressor-like cells (MDSCs-like) and 1 mast cells), 16 B cell clusters, 10 mesenchymal cell clusters (1 endothelial, 6 CAFs, and 3 myofibroblasts), 11 EPC clusters and 39T/NK cell clusters. Each cluster was composed of cells from different patients; and for each cluster, the distribution of cells with different tissue origins or with different treatment status was distinct.

### PC promotes the activation of B cells in the primary CRC

**Fig. 2:**
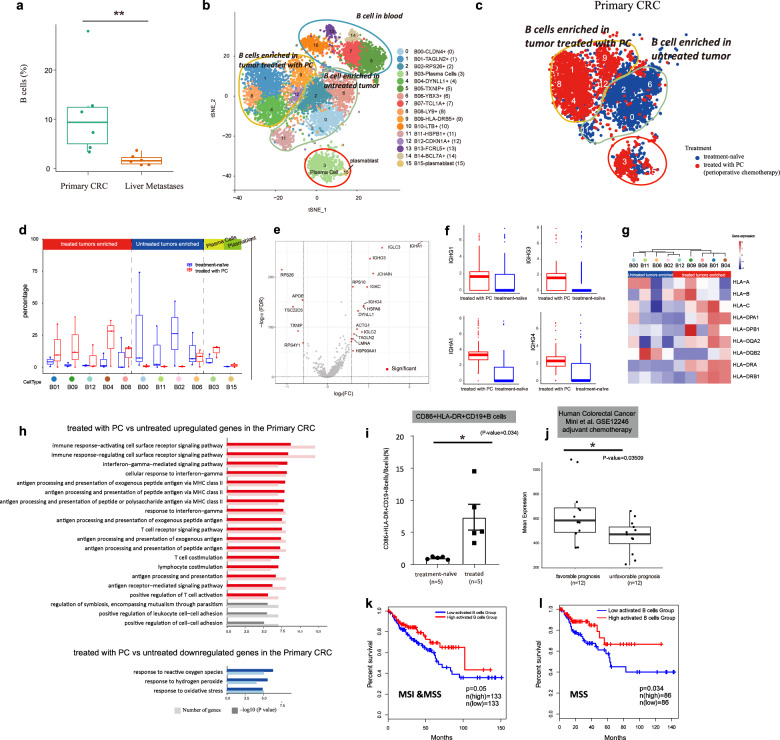
scRNA-seq of B cells reveals distinct subpopulation composition in tumors treated with PC or treatment-naïve. **a** Boxplot comparing the frequency of B cells between the primary CRC and liver metastases. Each dot represents the percentage of B cells from one sample. *P* value was calculated using Wilcoxon rank-sum test, **P < 0.01. **b** t-SNE plot of scRNA-seq profile from 7454 B cells separated into 16 subtypes. Cells are colored according to different cell types. **c** t-SNE plot of scRNA-seq profiles of B cells in the primary CRC. Cells are colored according to treatment status (treated with PC or without PC). **d** Boxplots showing the percentage of each B cell subcluster in untreated and treated samples in the primary CRC. The plot shows that B01, B04, B08, B09, and B12 are enriched in tumors treated with PC, whereas B00, B11, B02, and B06 are prevalent in untreated tumors. **e** Volcano plot showing DEGs between B cells enriched in tumors treated with PC and B cells prevalent in untreated tumors. Each red dot denotes an individual gene with adjusted *P* value < 0.05 and fold change ≥1.5 (two-sided moderated *t*-test with limma). **f** Box plots showing the expression of IGHG1, IGHG3, IGHG4, and IGHA1 in PC-treated and treatment-naïve tumors. **g** Heat map showing the relative expression of MHC molecules in each B cell subtype. **h** GO analysis for upregulated genes (top) and downregulated genes (bottom) with *P* < 0.05 in tumors treated with PC vs without PC. Pathways related to immune activation are colored by red; pathways related to inflammation are colored by blue. **i** The FACS results showing the abundance of activated B (CD86^+^HLA-DR^+^CD19^+^) cells in CRC patients treated with or without PC. **j** Boxplot showing the levels of the B cell signature in samples from pretreated colorectal cancer patients in the dataset GSE12246, adjuvant chemotherapy^86^. **k** The Kaplan–Meier overall survival curves of TCGA COAD patients grouped by different expression levels of signature genes of activated B cells enriched in PC-treated tumors. Patients involved in this analysis include MSI-high, MSI-low, and MSS patients. Patients separated by high- (*n* = 133) or low-level (*n* = 133) B cell gene signature exhibit different overall survival (*P* = 0.05). **l** Overall survival curves of TCGA COAD patients. Only MSS patients were involved in the analysis. *P* = 0.034.

First, we investigated how PC influenced the ability of B cells. In our single-cell data, B cells were relatively more abundant in primary CRC (account for 0.1% of all stromal cells in primary CRC), but depleted significantly in liver metastases (account for 0.01% of all stromal cells in liver metastases) (Fig. 2a). Sub-clustering of B cells revealed 16 subpopulations (Fig. 2b). Among them, 14 clusters were mature B cells (with nine subsets from tumor lesion and five clusters from peripheral blood, Fig. 2b), which are characterized by highly expression of CD20 (MS4A1). While cluster three represents plasma cells characterized by highly abundant immunoglobulin (IGHG1, IGHG2, IGHG3, IGHG4, and IGHA2), cluster 15 represents plasmablasts, characterized by upregulation of immunoglobulin and cell proliferation markers (e.g., MKI67, CDC20, CDKN3, and CCNB2) (Supplementary Table S3).

Since there were few B cells infiltrated in the liver metastases in our dataset, we mainly focused on B cells in the primary CRC. In primary tumor, B cells were separated into different subgroups, the B cell populations from tumor treated with PC were quite distinct from that in treatment-naïve tumors (Fig. 2c, d). We found that clusters 0, 2, 6, and 11 were enriched specifically in treatment-naïve tumors, whereas clusters 1, 4, 9, 8, and 12 were almost exclusively present in treated tumors (Fig. 2c, d).

On closer examination of the two groups, we found that they have distinct phenotypes (Supplementary Table S3). Untreated tumors-derived B cells exhibit a naïve and inflammatory phenotype, with cluster 6 expressing IgD (*IGHD*), cluster 0 expressing immature B cell marker VPREB3 and cluster 2 expressing inflammatory transcription factor NF-κB (*NFKBIA*), lipid molecules (e.g., APOE and APOC1), and cytokines (e.g., AREG), whereas B cells derived from treated tumors (clusters 1, 4, 9, 8, and 12) show an activated immune activation phenotype with the upregulation of immunocostimulatory molecules (e.g., CD82, CD83, and CLECL) and MHC molecules (e.g., HLA-DRA, HLA-DRB5) (Supplementary Table S3). Gene ontology (GO) analysis also confirms that upregulated genes associated with treated tumors are enriched in antigen processing and presentation (Supplementary Fig. S2a).

Compared with B cells in untreated tumors, higher expression of immunoglobulin, such as IGLC3, JCHAIN, IGHG1, IGHG3, IGHG4, and IGHA1, were observed in treated tumors (Fig. 2e, f and Supplementary Fig. S2b, Table S5), implying that class switch recombination (CSR, also known as isotype switching) may occur after PC. Moreover, the expression of MHC molecules (such as HLA-A, HLA-DQB1, HLA-DQA2, and HLA-DRB1) was also elevated in treated tumors (Fig. 2g). In line with these results, GO analysis showed that genes highly expressed in B cells in tumors treated with PC were enriched in immune activation-related signaling pathways or processes, including immune response-activating cell surface receptor signaling pathways, antigen processing and presentation, and T cell co-stimulation (Fig. 2h). In contrast, genes highly expressed in B cells in treatment-naïve tumors were enriched in the response to reactive oxygen species, a general feature of inflammation. Thus, these results support that the phenotype of B cells in treatment-naïve tumors is associated with inflammation. On the contrary, in treated tumors, PC treatment promoted the activation and the generation of class-switched antibodies in B cells. To validate the results and evaluate the clinical significance of the B cell signature obtained in this study, we solidified our findings using further experiments and also the published data from human clinical study. Flow cytometry verified that treated tumors presented a striking increase in activated B cells (Fig. 2i and Supplementary Fig. S2d). Representative multiplex immunofluorescence assay showed that activated B cells (HLA-DR^+^CD80^+^CD19^+^ B cells) were densely localized in tertiary lymphoid structures (TLSs) in treated patient, with only a few activated B cells localized in treatment-naïve patient (Supplementary Fig. S2c). The published data set of patients treated with chemotherapy also validated that the signature of B cells was enriched in cohorts of patients with favorable prognosis (Fig. 2j).

If B cells can contribute to anti-tumor processes, an effective immune response against tumor progress may be reflected by the presence of a gene expression signature of B cell activation. This led us to hypothesize that gene expression signatures of activated B cells may be correlated with the prognosis of CRC. To test this, we turned to The Cancer Genome Atlas (TCGA) colon adenocarcinoma (COAD) clinical data, and found that the gene signatures of activated B cells were associated with a good prognosis in CRC patients marginally significantly (Fig. 2k, *n* = 266, including microsatellite instability (MSI)-high, MSI-low and MSS subtype, *P* = 0.05, Cox regression). Interestingly, the correlation became more prominent in MSS tumors (Fig. 2l, *n* = 174, MSS subtype, *P* = 0.034, Cox regression), suggesting that activation of B cells could be more effective in MSS tumors.

The immunohistochemistry (IHC) results showed that B cells were densely localized in TLSs in primary CRC with only a few B cells infiltrated in liver metastases (Supplementary Fig. S2e), consisted with the observations in our single-cell RNA analyses (Fig. 2a). However, this finding needs further investigation in more samples.

Collectively, our data demonstrate that PC promotes the conversion of B cells from a less activated and inflammatory state to a more activated state in the primary tumor of CRC patients with liver metastases, and that the activation of B cells could be a potential predictor of effective chemotherapy and good prognosis, especially in patients with MSS CRC. This was also reported in some recent studies of tumors treated with immunotherapy^37–40^.

### PC promotes reprogramming of TAMs from high heterogeneity to immature and less activated phenotypes

**Fig. 3:**
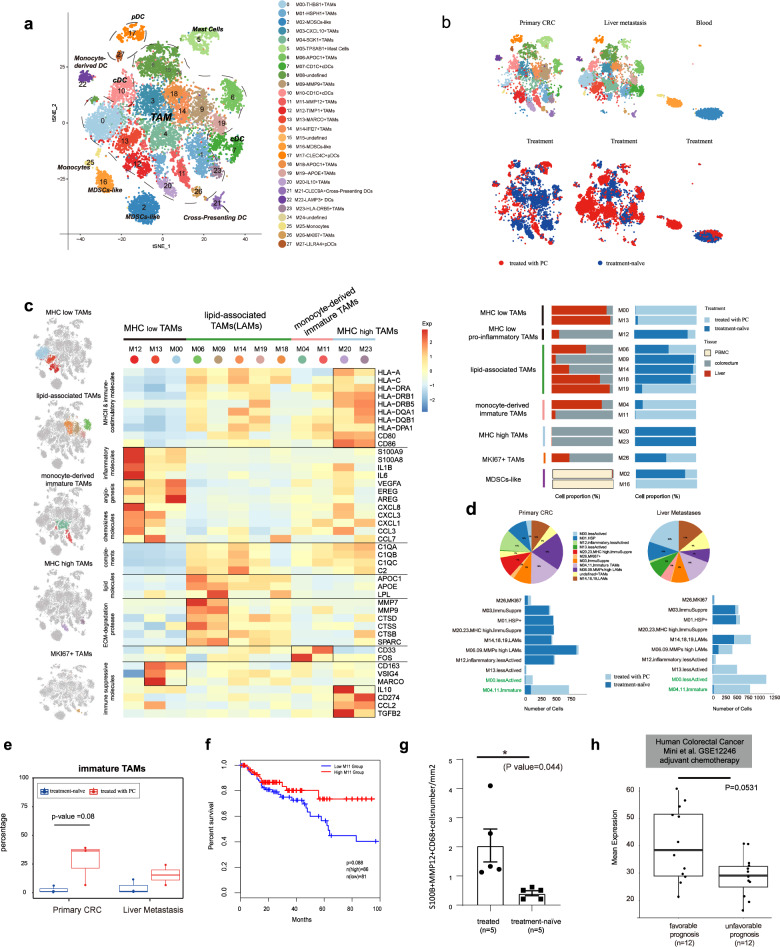
The phenotypic heterogeneity of myeloid cells. **a** t-SNE plot showing a total of 15,366 myeloid cells, separated into 28 subtypes. **b** Cells are colored according to tissue origins (top) and treatment status (bottom). **c** Left, heat map showing normalized expression (*z*-score) of function-associated genes in TAM subsets. Black boxes highlight the prominent patterns defining TAM subtypes. Right, bar plot showing the tissue origin and treatment status of each TAM subtype. **d** Bottom, based on the annotation and classification above, bar plots depicting cell numbers of each cell type in tumors with or without PC treatment are shown. Top, pie charts showing the proportions of different TAM subsets within different tissues (the primary CRC, liver metastases). **e** Boxplot comparing the frequency of immature TAMs in treated and treatment-naïve patients both in primary CRC and liver metastases. Wilcoxon rank-sum test was used for statistical analysis. **f** Overall survival curves of TCGA COAD patients (Cox regression). We identified an M11 TAM signature composed of 26 genes that showed significantly increased expression in M11 vs other TAM clusters. **g** Immunofluorescence analysis showing M11 (S100B^+^MMP122^+^CD68^+^) cell numbers in tumors treated with or without PC. **h** Boxplot showing the levels of M11 signature in pretreated samples from the human colorectal cancer dataset GSE12246, adjuvant chemotherapy^86^.

Myeloid cells are the key components in TME, with an important role in tumor progression and metastasis^41^. We identified 15,366 myeloid cells, sub-clustered into 28 clusters. Among these myeloid clusters, we designated 18 clusters as TAMs, which displayed various features. In addition, one cluster of monocytes (M25: *FCGR3A*), two clusters of MDSCs-like (M02 and M16), six clusters of DCs, including CD1C^+^ DCs (M07 and M10: *CD1C*), cross-presenting DCs (M21: *CLEC9A*), pDCs (M17 and M27: *LILRA4*) and LAMP3^+^ DCs (M22: *LAMP3*)^21^, and one cluster of mast cells (M05: *TPSAB1*) were identified. The expression of marker genes for the major lineages of myeloid cells was presented in Supplementary Fig. S3a. While TAMs and DCs were enriched in both primary tumor and liver metastases, monocytes and MDSCs-like cells were prevalent in blood and mast cells were mainly enriched in primary CRC (Fig. 3a, b).

M02 and M16 enriched the signature of MDSCs^42,43^. Consistent with the results reported by Zhang et al.^21^, S100A family genes are highly expressed, including S100A12, S100A9, S100A8, together with FCN1 and VCAN, but MHC I and MHC II molecules tend to be lowly expressed (Supplementary Table S3).

Macrophages commonly function as phagocytic cells, which can be activated and display varying phenotypes in response to different stimulations^44,45^. For the diversity and plasticity of TAMs, their heterogeneity and impacts on tumor progression remain largely uncharacterized^46^. In this study, we identified a total of 18 clusters of TAMs, among which three (M08, M15, and M24) were undefined clusters, which may represent low-quality clusters and were not included in further analyses. In TAMs, with no clear delineation between the phenotypes of M1 and M2. The M1 and M2 gene signatures are positively correlated in our TAM compartment (Supplementary Fig. S3b), indicating that TAMs were more complex than the classical M1/M2 model, consistent with previous studies^21,27,47–49^. Based on the transcription state and expressed genes of TAMs, we identified various signature genes and classified TAMs into four major TAM subsets (Fig. 3c).

The first subset, including clusters 0, 12, and 13, were classified as MHC^low^ TAMs. MHC^low^ TAMs exhibited weak capacity of antigen presentation and immune activation, with low expression of MHC I and MHC II genes (e.g., *HLA-A*, *HLA-C*, *HLA-DRA*, and *HLA-DRB1*) and immune-costimulatory genes (e.g., *CD80* and *CD86*). Cluster 12, classified as IL1B^+^MHC^low^ TAMs, was characterized by upregulation of a large number of inflammatory and chemokine genes (e.g., *IL1B*, *IL6*, *S100A9*, *S100A8*, *CXCL8*, *CXCL3*, and *CXCL1*) which involved in recruiting and regulating immune cells. Cluster 12 was present in the primary CRC of treatment-naïve patients (Fig. 3c, right panel, 3d). Cluster 0 (THBS1^+^MHC^low^ TAMs) was prevalent in liver (Fig. 3c, right panel), with high expression of *THBS1*, *MARCO* and genes promoting the proliferation of EPCs and angiogenesis, such as *EREG*, *AREG*, and *VEGFA*, which could stimulate tumor growth and progression. Cluster 13 (MARCO^+^ MHC^low^ TAMs), mainly derived from liver-resident Kupffer cells (the tissue-resident macrophages of liver), may exert a tolerogenic or immune inhibitory function in liver metastases through MARCO, VSIG4, and CD163.

The second subset, including clusters 6, 9, 14, 18, and 19, were classified as lipid-associated macrophages (LAMs), which were characterized by upregulation of genes involved in lipid metabolism (e.g., *APOC1*, *APOE*, and *LPL*), extracellular matrix (ECM) degradation (e.g., *MMP7*, *MMP9*, and *SPRAC*) and complement activation (e.g., *C1QA*, *C1QB*, and *C2*). They also highly expressed *TREM2* (encoding lipid receptor) and *LGALS3* (associated with immune suppression), which were recently found to be associated with metabolic diseases^50^. Among the subset, cluster 6 and cluster 9 showed higher expression levels of matrix metalloproteinases (MMPs) compared to other clusters. GO analysis of upregulated genes in each cluster demonstrated that they were enriched in neutrophil activation and degranulation (Supplementary Fig. S3c). In the primary CRC, LAMs were mainly present in treatment-naïve tumors, however, they were shared in liver metastases from both treated and untreated patients (Fig. 3c, right panel, 3d).

The third subset, including clusters 4 and 11, was enriched for genes involved in the regulation of myeloid leukocyte differentiation. They were prevalent in tumors treated with PC (Fig. 3c, right panel, 3d). With high expression of FOS, cluster 4, mainly from liver metastases, may be monocyte-derived, which could differentiate into macrophage^27^. In addition to the canonical myeloid marker gene *CD33*, cluster 11 also exhibited high expression of *CD4*, which is typically expressed by monocytes and involved in triggering cytokine expression and the differentiation of monocytes into functional mature macrophages^27,51,52^. Moreover, MHC genes tend to be expressed at a higher level in cluster 11 cells than cluster 4, suggesting that cluster 11 cells are more mature and activated than the latter. Thus, we classify this subset as monocyte-derived immature TAMs.

The fourth subset, including clusters 20 and 23, is immune-regulatory TAMs characterized by the upregulation of immune-suppressive genes, such as *CD274*, *CCL2*, *IL10*, and *TGFB2*, and are prevalent in primary CRC in treatment-naïve tumors (Fig. 3c, right panel). Strikingly, both of the two clusters exhibited the high expression of MHC and co-stimulating genes, a signature of immune activation and anti-tumor activities. Together, we uncovered “double-agent” immune regulatory TAMs with co-expression of both immune activated and immune suppressive genes, which indicate complex interactions between anti-tumor and pro-tumor activities.

In addition to the four subsets described above, there are three more clusters, M01, M03, and M26, that cannot be classified into the abovementioned four subsets. Genes highly expressed in these three clusters are associated with different biological processes and cellular functions. M26 (MKI67^+^ TAMs) is marked with high expression of genes involved in cell proliferation (e.g., *MKI67*, Supplementary Table S3). In addition to *EGF* and *MACRO*, heat-shock genes are also highly expressed in M01. M03 (CXCL10^+^ TAMs) highly expressed genes involved in response to interferon-gamma (*GBP1*, *STAT1*, *IFITM3*, and *PARP14*) and cellular response to zinc ion (*MT2A*, *MT1X*, and *MT1F*). Many phenotypes of TAMs are associated with the prognosis in CRC patients. The gene signatures of M12 and M20 are associated with good prognosis (*P* = 0.019 and *P* = 0.038, respectively), whereas the gene signature of M06 is associated with poor prognosis (*P* = 0.02) (Supplementary Fig. S3d).

TAMs from treatment-naïve tumors and chemotherapy-treated tumors exhibited distinct phenotypes in both primary tumor and liver metastases. In the primary CRC, TAMs in untreated tumors showed higher heterogeneity, however, they presented distinct phenotypes compared with TAMs in tumors treated with PC (Fig. 3d). Focusing on the treatment state, we identified some clusters shared between treated and untreated tumors, such as MKI67^+^ TAMs (M26). However, immature TAMs (M04, M11) and MHC^low^ TAMs (M00, THBS1^+^MHC^low^ TAMs) were largely specific to treated tumors (Fig. 3c, e). Instead, clusters of more activated TAMs (MHC^high^ TAMs, M20 and M23), MMPs^+^ LAMs (M06 and M09), pro-inflammatory TAMs (IL1B^+^MHC^low^ TAMs, M12), immune-suppressive TAMs (CXCL10^+^ TAMs, M03) and HSPH1^+^ TAMs (M01) were specific to treatment-naïve tumors (Fig. 3c, right panel, d). To quantify the heterogeneity of TAMs, we used Shannon’s Entropy to measure the diversity of TAMs phenotypes (see Materials and Methods section). The diversity of TAMs in untreated tumors (*y* = 2.81) is ~1.78 times of that of TAMs in tumors treated with PC (*y* = 1.58). In conclusion, PC promotes the reprogramming of TAMs from highly heterogeneity to immature and less activated phenotypes in primary tumors.

Concordant with observations in primary tumors, TAM populations with heterogeneous phenotypes were present in both treated and untreated tumors in liver metastases, including LAMs (M06, M09, M14, M18, and M19) and MKI67^+^ TAMs (M26). In contrast, MHC^low^ TAMs (M00, M13) and immature TAMs (M04, M11) were dominantly enriched in tumors treated with PC (Fig. 3d, e), while immune-suppressive TAMs (M03, CXCL10^+^ TAMs) and HSP^+^ TAMs (M01) were enriched in treatment-naïve tumors (Fig. 3d). Importantly, we found that the gene signature of M11 is associated with good prognosis marginally significantly (*P* = 0.088, Cox regression, Fig. 3f) in TCGA COAD patients within MSS subtype (*n* = 174), however, it shows little correlation with outcomes in MSI CRC patients, suggesting the infiltration of M11 could be a potential predictor of good prognosis in MSS CRC. Immunofluorescence analysis verified that the M11 (S100B^+^MMP12^+^CD68^+^ TAMs) cells were relatively more enriched in treated tumors (Fig. 3g and Supplementary Fig. S3f). Published dataset of patients treated with chemotherapy also validated that the expression of signature genes of M11 was much higher in the group with favorable prognosis (Fig. 3h). Trajectory analysis of myeloid cells showed that DCs and monocytes located at the origins of the trajectory axis, whereas TAMs were mainly enriched in the middle and differentiated ends (Supplementary Fig. S3g).

TAMs from tumors treated with PC are distinct from those in treatment-naïve tumors (Supplementary Fig. S3e) at the transcriptome level. In the niche of treatment-naïve tumors, TAMs from the primary CRC were enriched for genes involved in processes of neutrophil activation, response to IFN-γ and fibroblast proliferation (Supplementary Fig. S3e), indicating its proinflammatory phenotype^53^. TAMs from liver metastases were enriched for genes associated with antigen processing and presentation, neutrophil activation, and response to IFN-γ. In the ecosystem of treated tumors, TAMs in primary tumors were characterized by upregulated genes involved in the regulation of protein targeting to endoplasmic reticulum and RNA catabolic progress, while in the metastatic sites, TAMs were enriched for genes regulating myeloid leukocyte chemotaxis and migration, which might account for the aggregation of monocyte-like TAMs in this lesion.

In general, our results showed that PC suppressed the diversity of TAMs. After PC treatment, the majority of infiltrated TAMs in primary tumors were immature TAMs and THBS1^+^MHC^low^ TAMs, while more TAMs aggregated in liver metastases tended to be immature and less activated. Thus, chemotherapy facilitates the reprogramming of TAMs from high heterogeneity to immature and less activated phenotypes.

### PC decreases the abundance of ECM-remodeling CAFs, but promotes the accumulation of myofibroblast

**Fig. 4:**
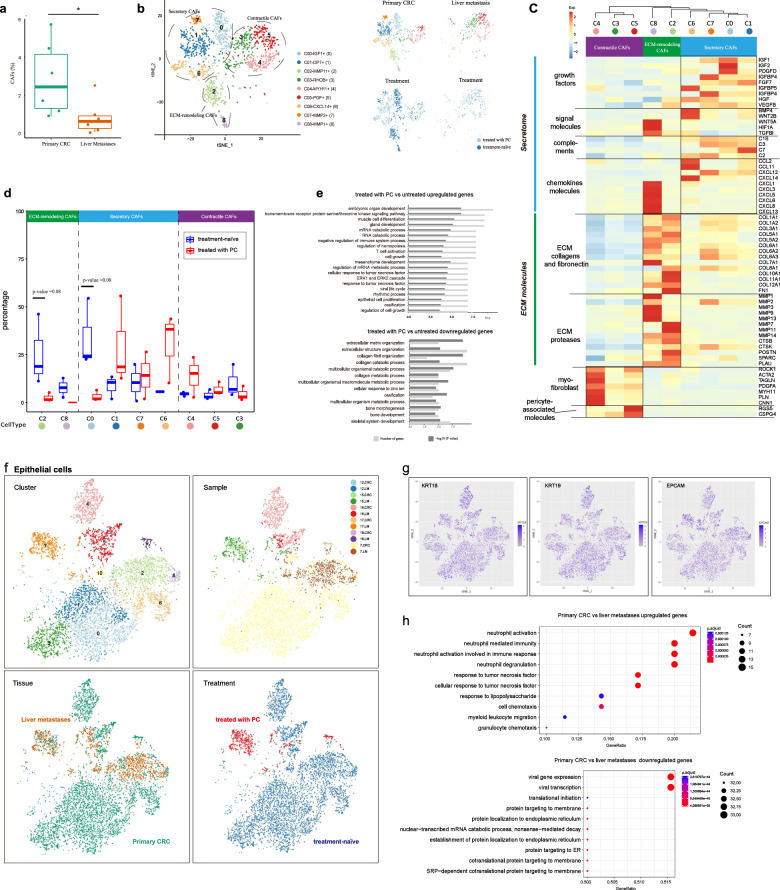
Compositions and phenotypes of CAFs and EPCs in primary CRC and liver metastases. **a** The percentage of CAFs in six primary CRC and six matched liver metastasis samples from six CRC patients with liver metastatic disease. Each dot represents one sample. *P = 0.031, two-sided Wilcoxon rank-sum test. **b** t-SNE plot showing a total of 1383 fibroblasts that can be separated into nine subtypes. Cells are colored according to different cell types (left), tissue origins (right, top), and treatment status (right, bottom). **c** Heat map showing the selected marker genes in each cluster. Relative expression was defined as the gene-wise (row) z-score of normalized UMI counts across CAF subtypes (column). **d** Box plots showing the percentage of each CAF subtype in primary CRC tumors treated with or without PC. Wilcoxon rank-sum test was used for statistical analysis. **e** GO analyses of genes that are differentially expressed between CAFs from tumors treated with PC and those from treatment-naïve tumors. Benjamini-Hochberg-corrected *P* values < 0.01. **f** Reclustering of EPCAM^+^ cells, colored according to clusters, sample origins, tissue origins, and treatment status. **g** t-SNE plots showing representative marker genes of EPCs. **h** GO analysis of genes that are differentially expressed between primary CRC and liver metastases in malignant cells. Selected GO terms with Benjamini-Hochberg-corrected *P* values < 0.05 (one-sided Fisher’s exact test).

In our study, 1383 CAFs were detected and classified into nine clusters. Notably, CAFs were significantly more abundant in primary CRC than in liver metastatic tumors (Fig. 4a). Based on the gene expression profile, we classified CAFs into three major subsets, including secretory CAFs (clusters 0, 1, 6, and 7), ECM-remodeling CAFs (clusters 2 and 8) and contractile CAFs (clusters 3, 4, and 5) (Fig. 4b, c). Secretory CAFs highly express secretory proteins, such as various growth factors (e.g., IGF1, PDGFD, FGF7, and VEGFB) that mediate angiogenesis and cancer cell proliferation, some signal molecules (e.g., BMP4 and WNT2B) that are able to maintain cancer stem cell niche, complements (e.g., C1S and C3) and chemokines (e.g., CCL2, CXCL12, and CXCL14) that regulate tumor immunity and inflammation. The ECM-remodeling CAFs highly express ECM proteins (such as ECM collagens and fibronectin), and are strongly associated with a fibrotic matrix (Fig. 4c). They also express a large number of ECM proteases, which alter ECM structure and assist tumor angiogenesis and metastasis^54^. The contractile CAFs are enriched for genes involved in the regulation of cell contraction (Fig. 4c), suggesting some distinct phenotypes. CAFs have numerous potential cellular sources. Cluster 4 exhibits myofibroblastic nature, as suggested by the upregulation of myofibroblast markers (e.g., ACTA2 and TAGLN) and genes involved in myogenesis (e.g., *MYH11*, *PLN*, and *CNN1*)^54,55^. Cluster 5, highly expressing pericyte-associated markers (e.g., RGS5 and CSPG4), largely originate from pericytes^54^. Cluster 3 exhibits upregulated expression of genes involved in stress response (e.g., *JUN*, *BAG3*, and *HSPA2*) and is only present in liver metastases, suggesting that they may be triggered as adaptation to TME.

CAFs derived from treated and untreated tumors exhibited distinct phenotypes (Fig. 4b, right panel). ECM-remodeling CAFs were prevalent in the primary CRC in treatment-naïve tumors (Fig. 4d), whereas contractile CAFs were more prevalent in tumors treated with PC both in primary tumors (cluster 4) and liver metastases (cluster 3 and cluster 5). Secretory CAFs were observed in the primary CRC, and were mainly enriched in treated tumors (except cluster 0) (Fig. 4d). Comparison of the CAFs from treatment-naïve tumors and treated tumors showed that CAFs from treatment-naïve tumors were strongly enriched for genes involved in processes of ECM organization and collagen metabolism (Fig. 4e), whereas CAFs in tumors treated with PC were significantly enriched for pathways involved in regulating muscle cell differentiation, immune system (T cell activation) and EPC proliferation (Fig. 4e). As we know, ECM remodeling is an important feature of CAFs common to progressive tumors and promotes metastasis^56^. The observations here indicate that PC suppresses ECM remodeling by CAFs, but promotes accumulation of myofibroblasts and diverse secretory CAFs in metastases of CRC.

Trajectory analysis of CAFs showed that the secretory CAFs, ECM-remodeling CAFs and contractile CAFs were enriched in branch 1, branch 2, and branch 3, respectively, confirming phenotypic distinction of the three subsets (Supplementary Fig. S3h). In addition, the pseudotime developmental trajectory showed that the secretory CAFs were considered as an earlier developed subtype. Contractile CAFs were highly enriched in the end of the trajectory axis, implying that they may be developed at the late stage of CAFs.

### EPCAM^+^ EPCs in TME

EPCAM^+^ EPCs were classified into 11 clusters, and were colored according to different clusters, sample origins, tissue origins, and treatment status (Fig. 4f, g). We confirmed that the EPCs were malignant by inferring chromosomal copy-number variations (CNVs) based on transcriptomes (see Materials and Methods section). Consistent with previous studies^19,57^, malignant cells show a patient-specific gene expression pattern. Interestingly, for each patient, malignant cells from different tissue origins (the primary CRC and the matched liver metastases) cluster together, reflecting that they have common origins (Fig. 4f, top right). After treated with chemotherapy, only a few EPCs were present in treated patients (mainly from COL15, see Fig. 4f) in our dataset. When comparing the transcriptomes of malignant cells in primary tumors and liver metastases, we noticed that a series of genes was especially expressed in the malignant cells of primary CRC but were absent in liver metastases. GO enrichment analysis revealed that these genes are enriched in immune-related processes, such as neutrophil activation involved in immune response, response to tumor necrosis factor (TNF), myeloid leukocyte migration, and granulocyte chemotaxis (Fig. 4h, top panel). This result suggests that cancer cells in the TME of liver metastases might present reduced immunogenicity, which allows them to easily escape immune detection.

### PC reduces CD8^+^ dysfunctional T cells

**Fig. 5:**
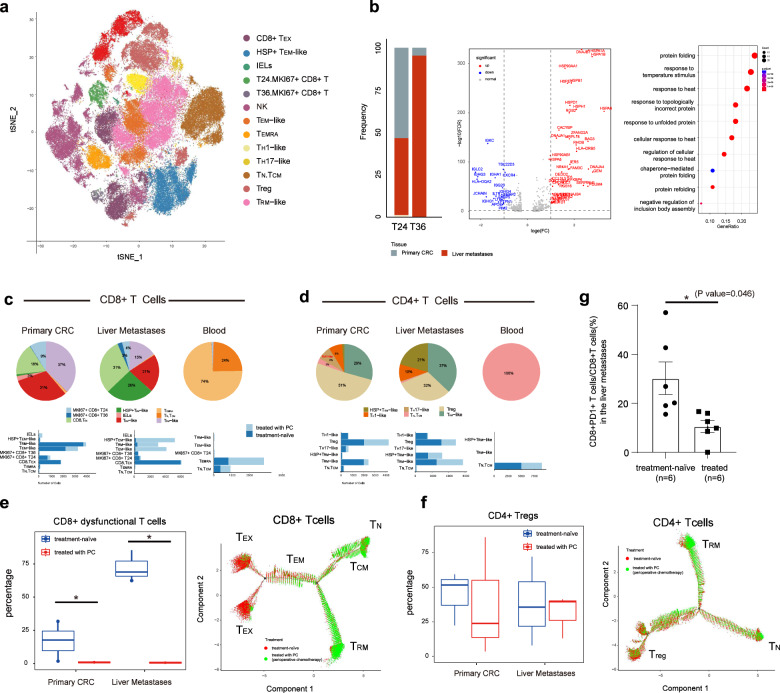
Cell subpopulations in the T cell compartment. **a** t-SNE plot showing a total of 86,803 cells classified into T/NK cell subtypes. **b** Bar plot exhibiting the distribution of T24 and T36 cells across different tissues (left). Volcano plot showing DEGs between the T24 and T36 clusters (middle). Each red/blue dot denotes an individual gene with fold change ≥ 2 and adjusted *P* value < 0.01 (two-sided moderated *t*-test with limma). GO analysis of DEGs between T24 and T36 (right). Selected GO terms with Benjamini-Hochberg-corrected *P* values < 0.05 (one-sided Fisher’s exact test) are shown. Bottom, based on the annotation and classification above, bar plots depicting cell numbers of each subtype of CD8^+^ T cells (**c**) and CD4^+^ T cells (**d**) in tumors with or without PC treatment are shown. Top, pie charts showing the proportions of different T cell subtypes within different tissues (the primary CRC, liver metastases, and blood). Left, frequencies of CD8^+^ dysfunctional T cells (**e**) and Tregs (**f**) in primary CRC and liver metastases with or without PC treatment are shown, respectively. Wilcoxon rank-sum test was used for statistical analysis. *P < 0.05. Developmental trajectory analysis of CD8^+^ T cells (**e**) and CD4^+^ T cells (**f**). Cells are colored according to the treatment states. **g** The FACS results showing the ratio of PD-1^+^CD8^+^ T cells to CD8^+^ T cells in liver metastases of CRC patients treated with or without PC.

Here, we also identified different phenotypes of T cells (Fig. 5a), including naïve T cells (T_N_), central memory T cells (T_CM_), intraepithelial lymphocytes (IELs), tissue-resident memory T cells (T_RM_)/effector memory T cells (T_EM_), recently activated effector memory T cells (T_EMRA_), dysfunctional or “exhausted” T cells (T_EX_), T_H_17-like cells, CXCL13^+^ T_H_1-like cells, MKI67^+^ T cells, and regulatory T cells (Tregs). Within these sub-populations, T_N_, T_CM_, and T_EMRA_ cells were mainly enriched in blood; IELs were most exclusively present in primary cancer; T_EX_ cells and T_RM_ cells were present both in primary and liver metastasis. The treatment state and tissue origin are mapped in Supplementary Fig. S4a. The annotation was confirmed by the expression of canonical markers (Supplementary Fig. S4b, c and Materials and Methods section).

In addition, we also identified two MKI67^+^CD8^+^ T cell populations (T36 and T24) (Fig. 5a). Closer examination of these two clusters revealed that cluster 36 was prevalent in the liver metastases (Fig. 5b, 96% in liver and 4% in the primary CRC), whereas cluster 24 was enriched in both the primary CRC and the liver (53% in CRC and 45% in liver), indicating that primary CRC and liver metastases share cluster 24, while the liver metastases have their specific T cells characterized by high proliferation activity (T36). To identify differences of these two subpopulations of T cells, we detected differentially expressed genes (DEGs) between T24 and T36, expressed by more than 10% cells, with the *P* value less than 1%, log_2_-fold change more than l. The results revealed that heat-shock protein, such as HSP90AA1, HSPA6, HSPA1A, HSPA1B, and DNAJA4, and some molecular chaperones (e.g., BAG3 and HSPB1) were greatly upregulated in cluster 36 (Fig. 5b). GO analysis showed that cluster 36 was mainly enriched for genes involved in protein folding and response to stress (e.g., temperature stimulus, heat, topologically incorrect protein), suggesting that they may be activated as adaptation to TME.

Many studies revealed that chemotherapy could impact on T cell diversity. For example, chemotherapy could increase tumor-infiltrating lymphocyte infiltration and decrease Treg accumulation and proliferation in CRC patients^58,59^. Comparing the cellular diversity of T cells in treatment-naïve tumors and tumors treated with PC, we found that most subsets of CD4^+^ T cells were shared in chemotherapy-treated and untreated tumors, however, the phenotypes of CD8^+^ T cells were significantly different (Fig. 5c, d).

In treatment-naïve tumors, different types of CD8^+^ T cells were present in the primary tumor, including effector T cells and exhausted T cells, while in the metastatic sites, for the CD8^+^ T cells, only dysfunctional or exhausted T cells were accumulated (Fig. 5c). Most significantly, PC inhibited the accumulation of dysfunctional T cells both in the niches of primary CRC and liver metastases (Fig. 5e). This was validated by flow cytometry (Fig. 5g and Supplementary Fig. S4d) and immunofluorescence analyses (Supplementary Fig. S5). Consistent with previous studies^58,59^, chemotherapy decreased the accumulation of Tregs in the primary CRC, but in liver metastases, the abundance of Tregs were comparable between treated and untreated tumors (Fig. 5f). The differentiation trajectories of CD8^+^ and CD4^+^ T cells (Fig. 5e, f and Supplementary Fig. S4e, f) also confirmed that the CD8^+^ dysfunctional T cells were most prevalent in treatment-naïve tumors, whereas the Tregs were shared in treated and untreated tumors. Dysfunctional CD8^+^ T cells are characterized by a loss of classical CD8 T effector function, such as cytotoxicity. The suppression of CD8^+^ dysfunctional T cells after chemotherapy may imply the reinvigoration of T cells.

### Cell–cell crosstalks within the TME in primary CRC and liver metastases

TME is a complex ecosystem. Cellular crosstalks determine tumor biology and response to therapies^60^. In order to systematically map cellular interactions especially those between different immune cells and mesenchymal cells in the TME of the primary CRC and metastases, and to investigate the potential cellular communications which contribute to cancer progression, metastasis, and immune evasion, we used CellPhoneDB v2.0.6 to study the crosstalks between stromal cells in TME. To visualize the crosstalks between different cells types, a chord diagram was built using the circlize package^61^ in R.

**Fig. 6:**
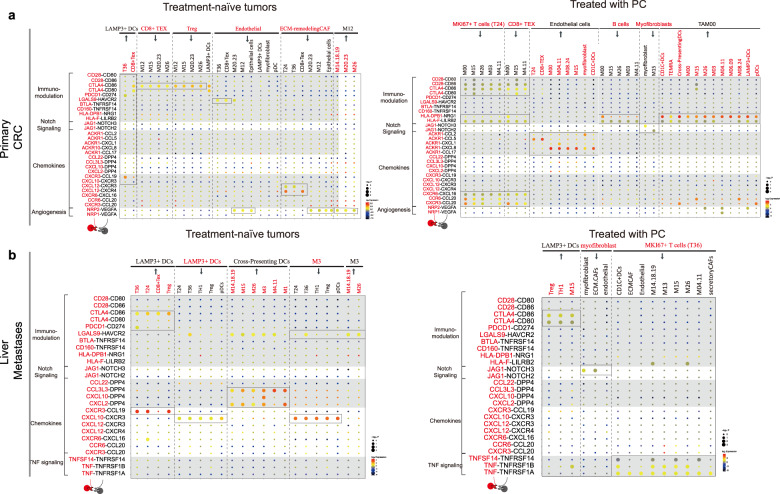
Cell–cell interaction networks in primary tumors and liver metastases of CRC. **a** Dot plot depicting the selected LR interactions enriched in treatment-naïve (left) and PC-treated (right) primary CRC tumors. Color intensity corresponds to the mean of average expression; dot size indicates the *P* values. Scales are shown on the right. **b** Dot plot depicting the selected LR interactions enriched in treatment-naïve tumors (left) and tumors treated with PC (right) in liver metastases. Two colors (red and black) are used to distinguish the cellular origin of each ligand/receptor.

First, we provided a landscape of crosstalks within major stromal cell types both in the niche of the primary CRC and the liver metastases. Then based on each cell type annotated above, we investigated intercellular communications in tumors treated with PC and untreated tumors. Selected LR pairs are summarized in Fig. 6, and the full list of results that were unique to different TME is available in Supplementary Table S4.

The crosstalk profiling of major stromal cells revealed that TAMs have the broadest crosstalks with other cells, both in the niches of primary CRC and liver metastases (Supplementary Fig. S6a, b). They display a rich LR profile, broadly communicating with mesenchymal compartment (CAFs and endothelial cells) and immune compartment (including T cells, NK cells, mast cells, DCs, and TAMs) in both the primary and liver metastases (Supplementary Fig. S6a). Comparison of the interactions in the primary CRC and liver metastases revealed that TAMs in the primary CRC communicate more frequently with CAFs than those in liver metastases. EPCs communicate more densely in the primary CRC, especially with TAMs, CAFs, and endothelial cells (Supplementary Fig. S6a), whereas the crosstalk between TAMs and DCs was greatly increased in the metastatic niche when compared with that in primary CRC (Supplementary Fig. S6a, b).

Based on the results above, we further specified key cellular interactions between cell subpopulations. The crosstalks between different subtypes of TAMs are most abundant in the TME, compared with other cell types. In addition, the crosstalks between subpopulations of DCs, CAFs, TAMs, dysfunctional T cells, and endothelial cells are relatively more frequent, whereas immune cells, such as B cells, plasma cells, other T cell subtypes (including T_EM_, T_RM_, T_EMRA_, and Tregs), NK cells and mast cells communicate less in TME (Supplementary Fig. S6c).

Moreover, compared with other T cell subtypes, we observed that MKI67^+^CD8^+^ T cells and T_EX_ display a rich LR profile (Supplementary Fig. S6c), and communicate densely with mesenchymal compartment (CAFs and endothelial cells) and immune compartment, indicating their immune-modulating functions. Both MKI67^+^CD8^+^ T cells and MKI67^+^ TAMs display a rich LR profile, implying that cells with high proliferative activity may communicate more with other cell types.

### Cell–cell communications in the niches of tumors treated with or without PC

Whether PC can reprogram the interactions within stromal cells is still unclear. To investigate the effects of chemotherapy on TME of liver metastases of CRC, we further compared cell*–*cell interaction networks between PC-treated and untreated tumors both in primary and metastatic sites.

Our data have revealed that the phenotypes of TAMs are highly heterogeneous, and the dysfunctional T cells and ECM-remodeling CAFs are enriched in the primary CRC of treatment-naïve tumors, while in the microenvironment of primary tumors treated with PC, chemotherapy promotes the activation of B cells and increases the abundance of immature and less activated TAMs and myofibroblasts.

The LR map showed that communications related to immune regulation were more frequent and broader in tumors treated with PC (Fig. 6a). In niches of treatment-naïve primary tumors, non-PC enriched TAMs (MHC^high^ TAMs (M20 and M23), MHC^low^ inflammatory TAMs (M12)) and MKI67^+^ TAMs (M26) expressed T cell immune checkpoint ligand CD86, and interacted with dysfunctional T cells and Tregs, directly inhibiting T cell function^62,63^ through LR pair CD86–CTLA4 (Fig. 6a, left). However, in the microenvironment of primary tumors treated with PC, the interactions involved in immune modulation were denser. For instance, PC-enriched TAMs expressed T cell immune checkpoint ligands CD86 and CD80, interacting with dysfunctional T cells and MKI67^+^ T cells through LR pair CD28–CD86, CD86–CTLA4, and CD80–CTLA4. Moreover, they mediate the release of chemokines CCL20 and CXCL16, recruiting CXCR3^+^CD8^+^ T cells, CCR6^+^ T cells, and CXCR6^+^ T cells through CCR6–CCL20, CXCR3–CCL20, and CXCR6–CXCL16 interactions (Fig. 6a, right).

Moreover, PC promoted the activation of B cells. In the niche of primary tumors treated with PC, B cells, MKI67^+^ T cells (T24) and dysfunctional T cells expressed HLA-F, and/or HLA-DPB1, interacting with PC-enriched TAMs through HLA-F–LILRB2 and HLA-DPB1–NRG1 interaction (Fig. 6a, right). Importantly, a subtype of PC-enriched TAMs (M0) also expressed NRG1 and immunomodulatory gene LILRB2, broadly interacting with DCs, T_EMA_ and other TAMs through HLA-F–LILRB2 and HLA-DPB1–NRG1 interactions (Fig. 6a, right). This implies the role of M0 involved in immune regulation.

Compared with the untreated tumors, the Notch signaling was exclusively activated in tumors treated with PC. The LR map in tumors treated with PC showed that myofibroblasts highly expressed JAG1, interacting with Notch receptors (NOTCH2, NOTCH3, and NOTCH4) on themselves and endothelial cells (Fig. 6a, right). Notch signaling regulates myofibroblast phenotype, tissue fibrosis^64^, and macrophage differentiation and functions^65^. In contrast, in the treatment-naïve tumors, we identified pro-angiogenic interactions among ECM-remodeling CAFs, non-PC-enriched TAMs, EPCs, and endothelial cells. MHC^high^ TAMs (M20 and M23) and inflammatory TAMs (M12) highly expressed pro-angiogenic factor vascular endothelial growth factor A (VEGFA), activating and recruiting ECM-remodeling CAFs and endothelial cells that might generate vascular networks in the microenvironment though NPR2–VEGFA^66,67^ (Fig. 6a, left). EPCs also expressed VEGFA, interacting with endothelial cells and ECM-remodeling CAFs through NRP1–VEGFA and NRP2–VEGFA in the primary CRC (Fig. 6a). Notably, among TAMs, high expression of VEGFA was found in inflammatory MHC^low^ TAMs (M12), which interacted with LAMs (M14, M18, and M19), MHC^high^ TAMs (M20 and M23), and MKI67^+^ TAMs (M26) through the NPR2–VEGFA interaction (Fig. 6a, left).

In addition, in tumors treated with PC, we found that endothelial cells densely communicated with other cells in the primary tumors. They express ACKR1 (DARC), broadly interacting with T cells (Supplementary Fig. S6d), DCs, myofibroblasts and TAMs through CCL5–ACKR1, CXCL8–ACKR1, CXCL1–ACKR1, and CCL17–ACKR1 interactions (Fig. 6a, right). ACKR1 plays a crucial role in regulating leukocyte recruitment^68^, and high expression of ACKR1 inhibits tumor growth, neovascularization, and metastasis^69^. In contrast, in untreated tumors, endothelial cells expressed immunosuppressive gene *LGALS9*, communicating with MKI67^+^ T cells (T36), MHC^high^ TAMs (M20 and M23) and dysfunctional T cells through the LGALS9–HAVCR2 interaction (Fig. 6a, left).

In liver metastases, chemotherapy promotes the abundance of DCs and myofibroblasts, but decreases the dysfunctional T cells. Both in tumors treated with and without PC, LAMP3^+^ DCs express CD86, CD274 (PDL1) and LGALS9, interacting with dysfunctional T cells, MKI67^+^ T cells and Tregs through CTLA4–CD86, PDCD1–CD274, and LGALS9–HAVCR2 interactions (Fig. 6b). However, in untreated tumors, LAMP3^+^ DCs further expressed CCL19 and CXCL10, recruiting CCR3^+^ Tregs, dysfunctional T cells and MKI67^+^ T cells through CXCR3–CCL19 and CXCR3–CXCL10 interactions (Fig. 6b, left).

In tumors treated with PC, in line with the observations in primary tumors, Notch signaling was also activated in liver metastases after chemotherapy (Fig. 6b, left). Importantly, TNF signaling was uniquely present in tumors treated with PC. MKI67^+^ T cells (T36) expressed TNF and TNFSF14, broadly interacting with immune cells (DCs and TAMs) and non-immune cells (CAFs) though TNF–TNFRSF1A, TNF–TNFRSF1B, and TNFSF14–TNFRSF14 interactions (Fig. 6b, right), which can positively regulate T cell response and contribute to the function of effector T cells as reported in previous studies^34,70^.

Compared with tumors after treatment, in treatment-naïve tumors, cross-presenting DCs expressed DPP4, interacting with TAMs through CXCL2–DPP4, CXCL10–DPP4, and CCL3L3–DPP4 interactions (Fig. 6b, left). DPP4 (also known as CD26) has been showed to be positively correlated with distant metastasis in CRC^71^, and CRC patients with high expression of DPP4 showed significantly worse overall survival^71^. In addition, cross-presenting DCs also interacted with TAMs through LGALS9–HAVCR2, implying their roles in immunosuppression.

In summary, our LR interaction map highlights that ACKR1, Notch signaling and molecules mediating immune regulation may contribute to the reprogramming of TME after PC treatment.

## Discussion

How to improve therapeutic options for patients with metastatic CRC is a core question for CRC treatment. In this study, we performed a single-cell profiling of primary CRC and their matched liver metastases with 111,292 cells, providing a fundamental and comprehensive understanding of cellular composition in TME of liver metastases of CRC. More importantly, for the first time, we provided a dynamic and comprehensive LR interaction mapping in stromal cells to illustrate how PC reprograms the TME of both primary CRC and matched liver metastases in CRC patients.

We find that B cells mainly exist in primary CRC. Importantly, our results indicate that PC may stimulate the activation of B cells. B cells in tumors treated with PC are characterized by high expression of Ig molecules (IgG and IgA) and MHC class II molecules, downregulation of naïve (e.g., VPREB3) and inflammatory markers (e.g., NFKBIA). Upregulation of IgG and IgA indicate that they undergo CSR, a key process in B cell activation and transformation into plasma cells when they are stimulated by antigens. The activation of B cells has also been found in the scenario of cancer immunotherapy^37–40^. Furthermore, we identified a gene signature of activated B cells and found that it is positively correlated with overall survival in the TCGA COAD cohort, especially in MSS subtype (Fig. 2i, j). Thus, our results suggest that the infiltration of the switched, activated B cells may play an important role in antitumor response, and that they could be a potential predictor of effective chemotherapy and good prognosis of CRC.

In this study, we classified TAMs into four major heterogeneous subclasses based on gene expression profiles (Fig. 3c). In the primary niche of treatment-naïve tumors, our results showed that hyper-inflammatory TAMs, MMPs^high^ TAMs, and MHC^high^ TAMs are prevalent in the primary CRC, whereas the abundance and diversity of TAMs decrease significantly after treatment with PC. According to previous studies, MHC^high^ TAMs are more likely to be activated TAMs, which are distributed in peritumor regions and contribute to tumor invasion and metastasis, resulting in shorter survival^72,73^. In addition, TAMs play a dominant role in tumor inflammation by facilitating angiogenesis and promoting tumor growth and metastasis^74–77^. The subset of inflammatory TAMs exhibit a strong inflammatory phenotype and may significantly contribute to tumor progression. Taken together, TAMs in TME of treatment-naïve tumors may promote tumor development. In contrast, characteristics of TAMs in tumors treated with PC are quite different. We found that immature and less activated TAMs are more enriched in treated tumors. Especially, the gene signature of immature TAMs (M11) is associated with better prognosis in MSS cohort. According to recent studies, high TAM density is closely correlated with poor survival in many cancers^78,79^. However, TAMs-targeted therapy has not been effective yet, which may be hampered by TAM heterogeneity and elusive molecular phenotypes. Our study provides a full-scale illustration of TAM composition and molecular characteristics, which is an important foundation and resource for TAMs-targeted therapy research to inhibit and cure metastatic CRC. Our results also reveal that PC suppresses the abundance of dysfunctional T cells and ECM-remodeling CAFs, and induces the generation of myofibroblasts. The accumulation of myofibroblasts indicates tissue injury and fibrosis related to chemotherapy.

**Fig. 7:**
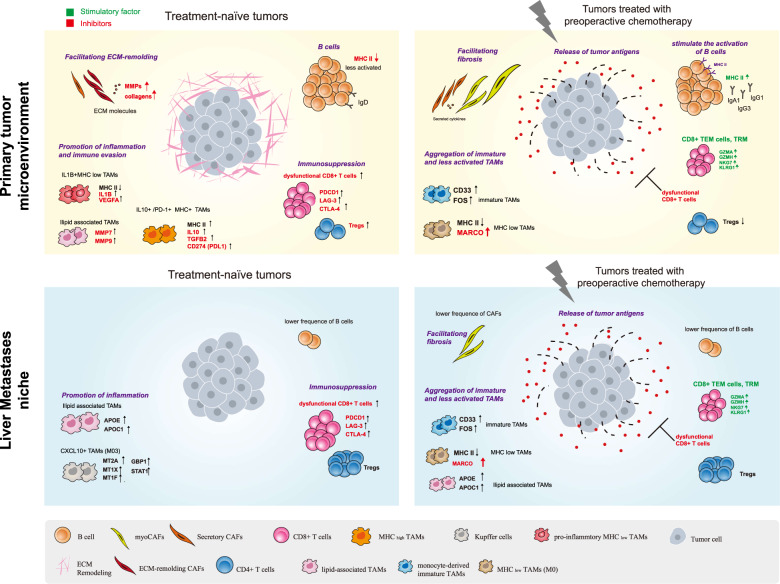
**Diagram illustrating the reprogramming of TME in response to PC.** The reprogramming of TME in response to PC. Top, schematic diagram of the reprogramming of TME in treatment-naïve (left) and treated (right) tumor in the primary CRC. Bottom, schematic diagram of the reprogramming of TME in treatment-naïve (left) and treated (right) tumor in liver metastases.

Therefore, based on the phenotypic alteration that we observed in the primary CRC treated with or without PC, we deduced that chemotherapy destroys tumor cells, which releases tumor antigens and activates immune microenvironment (Fig. 7), including B cell maturation and antibody generation. Moreover, chemotherapy reduces the diversity of TAMs and remodels the characteristics of TAMs, converting inflammatory TAMs into an immature and less activated phenotype. However, the TME of liver metastasis is clearly different. The number of B cells in the liver metastasis shows a significant reduction, which might be a cause for the feasibility of liver metastasis of CRC. Similar to the situation of primary CRC, myofibroblasts are aggregated in response to chemotherapy, which leads to fibrosis of liver metastases.

Taken together, this atlas provides a fundamental reference for future studies of the complex cellular and phenotypic diversity within TME of both primary CRC and liver metastases. Our systematic investigation of transcriptional changes and phenotypic alteration in TME at single-cell level may provide valuable insight into our understanding of therapeutic outcome. This may open up new possibilities to develop or improve therapeutic strategies for CRC.

## Materials and Methods

### Tumor specimens and patient clinical characteristics

Primary CRC, matched liver metastases and blood samples were collected from six CRC patients with metastatic disease. All patients were classified as MSS with invasive adenocarcinomas and late-stage (IV) disease (Supplementary Table S1). For each lesion, we collected the tissue in the core of the tumor after the surgery. Single cells were isolated from fresh tumor tissues without surface marker pre-selection. All patients underwent curative intent surgery of synchronous colectomy with liver resection. In addition, all patients who provided specimens signed an informed consent form and agreed to the specimens being used for scientific research. Detailed pathological and clinical information of patients is listed in Supplementary Table S1. Among the patients, patients COL15, COL 17, and COL18 were treated with PC, and the others were treatment naïve. Among patients treated with PC, patient COL15 received three cycles of CAPEOX (capecitabine plus oxaliplatin) on weeks 1, 5, and 9. Patient COL17 received four cycles of CAPEOX on weeks 1, 5, 9, and 13. Patient COL18 received eight cycles of FOLFOX-Bev (5FU, oxaliplatin, leucovorin with bevacizumab), 2 weeks per cycle. Surgery was performed ~1 month after the last chemotherapy treatment in all three patients. All three patients in this study responded well to PC with a significant tumor shrinkage. For more details, please see Supplementary Table S1.

### Tumor disaggregation and single-cell collection

Venous blood was collected before surgery in EDTA anticoagulant tubes and used to isolate PBMC immediately. Fresh biopsies of the primary CRC and the matched liver metastases were collected during surgery. Once the specimens were separated from body, they were processed for scRNA-seq immediately.

### PBMC Isolation

PBMCs were isolated using Ficoll (TBD) solution according to the manufacturer’s instructions. In brief, 5 mL fresh peripheral blood was layered onto equal Ficoll, following by centrifugation at 450 × *g* for 25 min. After centrifugation, lymphocyte layer remained at the plasma-Ficoll interface and were carefully transferred to a new tube and washed twice with phosphate-buffered saline (PBS, ThermoFisher Scientific). Lymphocyte pellets were re-suspended with sorting buffer (Hank’s Balanced Salt Solution (HBSS, ThermoFisher Scientific) with 0.04% bovine serum albumin (BSA, MRC)) for flow cytometry analyses.

### Tumor Dissociation

The primary CRC and metastatic tumor tissue were dissociated using MACS^®^ Tumor Dissociation Kit (Miltenyi Biotec). Briefly, Fresh biopsy samples of the primary and metastatic tumors were washed with Dulbecco’s PBS (ThermoFisher Scientific), minced into ~1-mm^3^ pieces, and enzymatically digested with Human Tumor Dissociation Kit (Miltenyi Biotec) for 60 min on a rotor at 37 °C, according to the manufacturer’s protocol. Cell suspension was subsequently filtered through a 40-μm Cell Strainer (BD) and centrifuged for 10 min at 400× *g*. The supernatant was then removed, pelleted cells were suspended in red blood cell lysis buffer (Solarbio) and incubated on ice for 2 min to lyse red blood cells. After washing with HBSS, the cell pellets were re-suspended in sorting buffer (HBSS with 0.04% BSA) for flow cytometry process.

### Sorting of viable single cells

A single-cell suspension was stained for viability with 1 μm Calcein AM (ThermoFisher Scientific) and 0.33 μM TO-PRO-3 iodide (ThermoFisher Scientific) prior to sorting. Fluorescence-activated cell sorting was performed on BD Influx (BD Biosciences) using 488 nm (calcein AM, 530/40 filter), 638 nm (TO-PRO-3, 670/30 filter) lasers. Singlets were captured and doublets were discarded through forward scatter height and width parameters. Viable cells were recognized as Calcein AM (high) and TO-PRO-3 (low) cell cluster. For PBMC sample, only lymphocyte and monocyte clusters were selected for further sorting. Viable single cells were resuspended in HBSS with 0.04% BSA. Viability was confirmed to be > 90% using trypan blue (ThermoFisher Scientific) exclusion prior to scRNA-seq process.

### Droplet-based scRNA-seq and library preparation

The scRNA-seq libraries were constructed by using the Chromium™ Single Cell 3′ Reagent Kits v2 (10× genomics) according to the manufacturer’s instruction. Briefly, cells were suspended in HBSS with 0.04% BSA at a concentration ~1000 cells/μL and appropriate suspension loading volume were determined by calculating for a target capture of 8000 cells. Cell suspension of corresponding volume was loaded onto the 10× Chromium Single Cell Platform (10× genomics). Generation of gel beads in emulsion (GEMs), barcoding, GEM-RT clean-up, complementary DNA amplification and library construction were all performed according to the manufacturer’s protocol. Sequencing library quality was checked with Bioanalyzer (Agilent Bioanalyzer 2100). Library quantification was measured using Qubit before pooling. The final library pool was sequenced on the Illumina NovaSeq 6000 instrument using 150-base-pair paired-end reads. Sequencing data of individual samples were summarized in Supplementary Table S2.

### Preprocessing of scRNA-seq data analysis

The raw base call (BCL) files were demultiplexed into FASTQ file by bsl2fastq. Droplet-based sequencing data were qualified by FastQC software. Then reads were aligned against GRCh38 human reference genome provided by Cell Ranger (version 2.0, 10× genomics), unique molecular identifier (UMI) counts were summarized for each cell of each gene. The raw UMI count matrices were converted into a Seurat object by the R package Seurat^80^ (version 2.3.4) and then filtered to (1) remove cells with a low number of unique detected genes (< 500); (2) for each batch, remove cells for which the total number of UMI (after log_10_ transformation) was not within the three standard deviations of the mean; (3) for each batch, remove cells that showed an unusually high or low number of genes; (4) discard cells in which the proportion of the UMI count attributabled to mitochondrial genes was greater than 15%. Overall, 731 cells were filtered out in step 1, while step 2 to step 4 removed only a small number of cells (0.1%). After exclude low-quality cells, 25,121 protein-coding genes across 111,292 single cells remained for downstream processing.

### Identification of cell types and subtypes by dimensional reduction

After quality control, raw UMI counts were lognormalized using the scale of 10,000. The genes with normalized expression between 0.0125 and 3, and dispersion > 0.5 were selected as highly variable genes. 1511 highly variable genes were identified based on dispersion and mean. “var.to.regress” option UMI’s and percent mitochondrial content were used to regress out unwanted sources of variation. The resultants were first summarized by principle component analysis. We used the function *FindClusters* on 50 principle components with resolution 1.0 to perform the first-round cluster and annotation. The annotation of each cell cluster was confirmed by the expression of canonical marker genes. As shown in Supplementary Fig. S1b, EPCs were identified using the higher expression of EPCAM, and other cell types were annotated using: T cells (CD3D, CD3G, TRAC), B cells (CD19, CD79A, and MS4A1), plasma cells (IGHG1, IGHA1, MZB1, and CD79A), monocytes and macrophages (CD68, CD163, CD14, and LYZ), NK Cells (KLRF1, KLRD1, FGFBP2, and PRF1). CAFs (FAP, COL1A1, COL3A1, DCN, and ACTA2), endothelial cells (CLDN5, CDH5, and VMF), pDC (LILRA4 and IL3RA), and mast cells (TPSAB1, TPSB2, and MS4A2). Then focusing on each major cell types, the same clustering protocol was used to identify clusters within the major cell types aforementioned.

Among T cells, cell clusters were identified using genes previous reported. Naïve T cells were identified by the expression of “naïve” marker genes, such as CCR7, SELL, and LEF1. T_RMRA_ cells were identified by the expression of cytotoxic markers KLRG1, GZMH, NKG7, and PRF1, but without upregulation of inhibitory molecules. T_H_17-like cells exhibit upregulation of IL23R and IL17A, and T_RM_-like cells express markers CD69, IL7R, CXCR4, and GPR18. T_EM_ clusters were characterized by high expression of chemokine receptor CXCR4 and mild expression of cytotoxic molecules (GZMK and IFNG). Tregs are marked by high expression of FOXP3 and IL2RA. Moreover, a small subset of T cells were characterized as IELs based on the highly expressed γδT cell receptors (TRGC2) and NK cell markers. Furthermore, consistent with previous results^22,34^, a subset of follicular helper T cells (CXCL13^+^ T_H_1-like cells) was observed in CD4^+^ T cells, highly expressing CXCL13 and some inhibitory molecules (such as CTLA-4).

### Pathway enrichment analysis

We used the R package limma^81^ to identify DEGs between cells from treatment-naïve tumors and chemotherapy-treated tumors. The Benjamini-Hochberg multiple testing correction was applied to estimate the FDR.

Biological-process GO enrichment (*P* < 0.01) was performed using clusterProfiler packages (version 3.9.2)^82^ with a Benjamini–Hochberg multiple testing adjustment. Gene sets with a FDR-corrected *P* < 0.01 were considered to be significantly enriched.

### Statistical analysis

All statistical analyses were conducted using R software (R Foundation for Statistical Computing). Statistical analysis data were presented as the means ± SEM of three independent experiments. Comparisons between two groups of samples were evaluated using Wilcoxon rank-sum test (Mann–Whitney U-test) for statistical analysis. *P < 0.05, **P < 0.01, ***P < 0.001.

### The phenotypic diversity of TAMs

To quantify the heterogeneity of TAMs, we used Shannon’s Entropy to measure the diversity of TAMs phenotypes. For each phenotype, we calculated the proportion (*p*) of cells coming from each phenotype among all TAM cells. The phenotypic diversity of TAMs is then calculated based on Shannon’s Entropy:

$$y = - \mathop {\sum }\nolimits_{n = 1}^n p(xn)\log _2[p(xn)]$$$$p(xn)$$ is the frequency of the number of cells with the phenotype *n* in the niche of TAMs, and $$\mathop {\sum }\nolimits_{n = 1}^n p\left( {xn} \right) = 1$$.

### Estimation of CNVs in cancer cells

The InferCNV package^83^ was used to detect the CNVs in EPCAM^+^ cells and to recognize real cancer cells with the following parameters: “denoise”, default hidden Markov model settings, and cutoff = 0.1. The chromosomal expression patterns were estimated from the moving averages of 101 genes as the window size and adjusted as centered values across genes. Two clusters mainly containing non-malignant derived cells were used as the control group.

### Putative interactions between cell types

We used CellPhoneDB v2.0.6 (www.cellphonedb.org) to study the crosstalk between stromal cells in TME. CellPhoneDB is a repository of curated receptors, ligands, and their interactions to predict communicating pairs. It integrates multiple databases and includes subunit architecture for both ligands and receptors to represent heteromeric complexes accurately^18,84^.

In brief, a count file containing gene expression value and a meta file with the cell type annotation information were prepared as inputs to the algorithm. Pairwise cell*–*cell interaction analyses were performed by CellphoneDB. In our data, only ligands or receptors expressed in more than 10% of the given cell subpopulations were considered as potential candidates. The interactions between subpopulations were identified as follows. (1) Randomly permuted the labels of all cells 1000 times, then determine the mean of the average receptor expression level in a cluster and the mean of the average ligand expression in their counterpart clusters. Thus, a null distribution could be obtained for each LR pair in each pairwise comparison. (2) Calculate the proportion of the means. If the means were the same as or higher than the actual mean, a *P* value for the likelihood of cell-type specificity of a given LR complex was obtained. (3) Selected LR pairs that have significant *P* values and are biologically relevant. To visualize the crosstalk between different cells types, a chord diagram was built using the circlize package^61^ in R.

### Tajectory analysis

We used Monocle2^85^ to construct the cell differentiation trajectories. The dimensionality reduction was performed with the DDRTree algorithm, using the most highly variable genes (top 1000) to arrange the cells in order. Genes which changed along the identified trajectory were identified by performing a likelihood ratio test using the function “differentialGeneTest” in the monocle package. The minimum spanning tree on cells was plotted by the visualization functions “plot_cell_trajectory” or “plot_complex_cell_trajectory”. BEAM tests were performed on the first branch points of the cell lineage using all default parameters. “Plot_genes_branched_pseudotime” function was performed to plot a couple of genes for each lineage.

### TCGA data analysis

The TCGA COAD data were used to evaluate the correlation between selected gene signatures and patient survival. The gene expression data were downloaded from UCSC Xena (http://xena.ucsc.edu/), clinical data were download from the Genomic Data Common Data Portal (https://gdc-portal.ncu.nih.gov/). The statistical analysis was performed by the R package “survival”, the survival curved were filtered by *survfit* function. The feature genes used for B cell signature were based on the differentially expression genes (FDR < 0.01, FC > 1.5) of the B cells from tumors treated with PC vs the B cells from treatment-naïve tumors. The genes used for immature TAMs signature were DEG among all TAMs subsets.

### Multicolor immunohistochemistry (IHC)

Multicolor IHC staining of formalin-fixed, paraffin-embedded (FFPE) tissue sections was used to confirm the presence of novel subpopulations, including dysfunctional T cells (PD-1^+^CD8^+^CD3^+^ T cells), M11 TAMs (S100B^+^MMP12^+^CD68^+^ TAMs), activated B cells (HLA-DR^+^CD80^+^CD19^+^ B cells), and validate the potential physical interaction (co-localization) between ACKR1^+^ endothelial (ACKR1^+^CD31^+^ endothelial cells) and CCL5^+^ T cells (CCL5^+^CD8^+^CD3^+^ T cells). Multicolor IHC staining was performed using PANO 7-plex IHC kit (0004100100, Panovue). Briefly, FFPE tissue sections (4 μm) were melted at 60 °C for 1 h followed by deparaffinizing and rehydrating. Heat-mediated antigen retrieval was performed in citrate acid buffer (pH 6.0) using microwave incubation. The sections were blocked with blocking buffer (hydrogen peroxide) for 10 min. The primary antibodies used in the validation of the novel subpopulations (including dysfunctional T cells (PD-1^+^CD8^+^CD3^+^ T cells), M11 TAMs (S100B^+^MMP12^+^CD68^+^ TAMs) and activated B cells (HLA-DR^+^CD80^+^CD19^+^ B cells)) were: anti-PD-1 (ZM-0381, Zsbio), anti-CD8 (ZM-0508, Zsbio), anti-CD3 (85061, CST), anti-S100β (ET1610-3, Huabio), anti-CD68 (ZM-0060, Zsbio), anti-MMP12 (ET1602-42, Huabio), anti-HLA-DR (97971, CST), anti-CD19 (ET1702-93, Huabio), and anti-CD80 (ET1702-95, Huabio). The primary antibodies used in the validation of the potential physical interaction (co-localization) between ACKR1^+^ endothelial cells and CCL5^+^ T cells were: anti-ACKR1 (137044, abcam), anti-CD31 (ET1608-48, Huabio), anti-CCL5 (ET1705-70, Huabio), anti-CD8 (ZM-0508, Zsbio), and anti-CD3 (85061, CST). After successive washes for ~3 times, sections were incubated for 10 min at room temperature with an anti-rabbit or anti-mouse horseradish peroxidase-conjugated secondary antibody (0004100100, Panovue). Each of the antibodies was connected with one fluorophore to detect antibody staining. The stained signals were further amplified using PPD520, PPD540, PPD570, PPD620, PPD650, PPD690, tyramide signal amplification (TSA) reagents through incubated with TSA diluent (0004100100, Panovue). To avoid spectral overlap between the fluorophores used in each panel, excitation wavelengths used were kept at a certain distance. Finally, nuclei were stained with DAPI.

### Multispectral imaging

The obtain multispectral images, the stained slides were scanned using the Mantra system (Perkin Elmer), which captures the fluorescent spectra at 20-nm wavelength intervals from 420 to 720 nm with identical exposure time. The scans were combined to build a single stack image. Images of unstained and single-stained sections were used to extract the spectrum of autofluorescence of tissues and each fluorescein, respectively. The extracted images were further used to establish a spectral library required for multispectral unmixing by InForm image analysis software (PerkinElmer). Using this spectral library, we obtained reconstructed images of sections with the autofluorescence removed.

### Flow cytometry analysis

Tissue samples were disassociated as described above. CD19^+^HLA-DR^+^CD86^+^ B cells were collected by flow cytometry. The following antibodies were used: anti-human CD45 conjugated to PE-cy7 (clone HI30, 304016, Biolegend), anti-human CD19 conjugated to Brilliant™ Violet 650 (BV650) (clone SJ25C1, 563226, BD Biosciences), anti-human CD3 conjugated to fluorescein isothiocyanate (FITC) (clone UCHT1, 555332, BD Biosciences), anti-human HLA-DR conjugated to APC (clone G46-6, 559866, BD Biosciences), and anti-human CD86 conjugated to PE (clone IT2.2, 555665, BD Biosciences). Anti-CD45 and anti-CD19 were used at 1:40 dilution. Anti-CD3, anti-HLA-DA, and anti-CD86 were used at 1:10 dilution. Fixable Viability Dye eFluor™ 780 (FVD780) (65-0865-18, ThermoFisher) was used to label dead cells and was used at 1:1000 dilution. For the CD8^+^PD-1^+^T cells, antibodies used included anti-human CD3 conjugated to FITC (clone UCHT1, 555332, BD Biosciences), anti-human CD4 conjugated to Brilliant™ Violet 510 (BV510) (clone SK3, 562970, BD Biosciences), anti-human CD8 conjugated to Brilliant™ Violet 605 (BV605) (clone SK1, 564116, BD Biosciences), anti-human PD-1 conjugated to Alexa Fluor^®^647 (clone MOPC-21,400130, Biolegend), isotype antibody conjugated to AF647 (clone MOPC-21, 400130, Biolegend). Anti-CD4, anti-CD8, anti-PD-1, and two isotype antibodies were used at 1:40 dilution. Anti-CD3 was used at 1:10 dilution. Fixable Viability Dye eFluor™ 780 (FVD780) (65-0865-18, ThermoFisher) was used to label dead cells and was used at 1:1000 dilution. Data analysis was performed in FlowJo (V10).

## Supplementary information

Supplementary information

Supplementary Table S1

Supplementary Table S2

Supplementary Table S3

Supplementary Table S4

Supplementary Table S5

## Data Availability

Raw data from scRNA-seq analysis have been deposited in the NCBI Gene Expression Omnibus (GEO) under accession numbers GSE178318. Source Data for Figures and Supplementary Figures are provided within the online content of this paper.
