# Author Correction: A single-cell atlas of liver metastases of colorectal cancer reveals reprogramming of the tumor microenvironment in response to preoperative chemotherapy

**DOI:** 10.1038/s41421-023-00537-z

**Published:** 2023-03-22

**Authors:** Li-Heng Che, Jing-Wen Liu, Jian-Ping Huo, Rong Luo, Rui-Min Xu, Cai He, Yu-Qing Li, Ai-Jun Zhou, Piao Huang, Yong-Yu Chen, Wen Ni, Yun-Xia Zhou, Yuan-Yuan Liu, Hui-Yan Li, Rong Zhou, Hui Mo, Jian-Ming Li

**Affiliations:** 1 Department of Pathology, Sun Yat-Sen Memorial Hospital, Sun Yat-Sen University, Guangzhou, Guangdong China; 2 Guangdong Provincial Key Laboratory of Malignant Tumor Epigenetics and Gene Regulation, Sun Yat-Sen Memorial Hospital, Sun Yat-Sen University, Guangzhou, Guangdong China

**Keywords:** Cancer microenvironment, Colorectal cancer, Chemotherapy

Correction to: *Cell Discovery* (2021) 7:80

10.1038/s41421-021-00312-y published online 07 September 2021

The original version of this article unfortunately contained a mistake. The name of Rui-Ming Xu is incorrect, and the correct name is Rui-Min Xu. We apologize for any inconvenience that it may have caused.

