# Supplementary material for: A single-cell atlas of liver metastases of colorectal cancer reveals reprogramming of the tumor microenvironment in response to preoperative chemotherapy
Source: Cell Discov. 2021 Sep 7;7:80. doi: 10.1038/s41421-021-00312-y (PMC8421363; doi:10.1038/s41421-021-00312-y)
Supplement: Supplementary file 1 — Supplementary information [file 41421_2021_312_MOESM1_ESM.pdf]

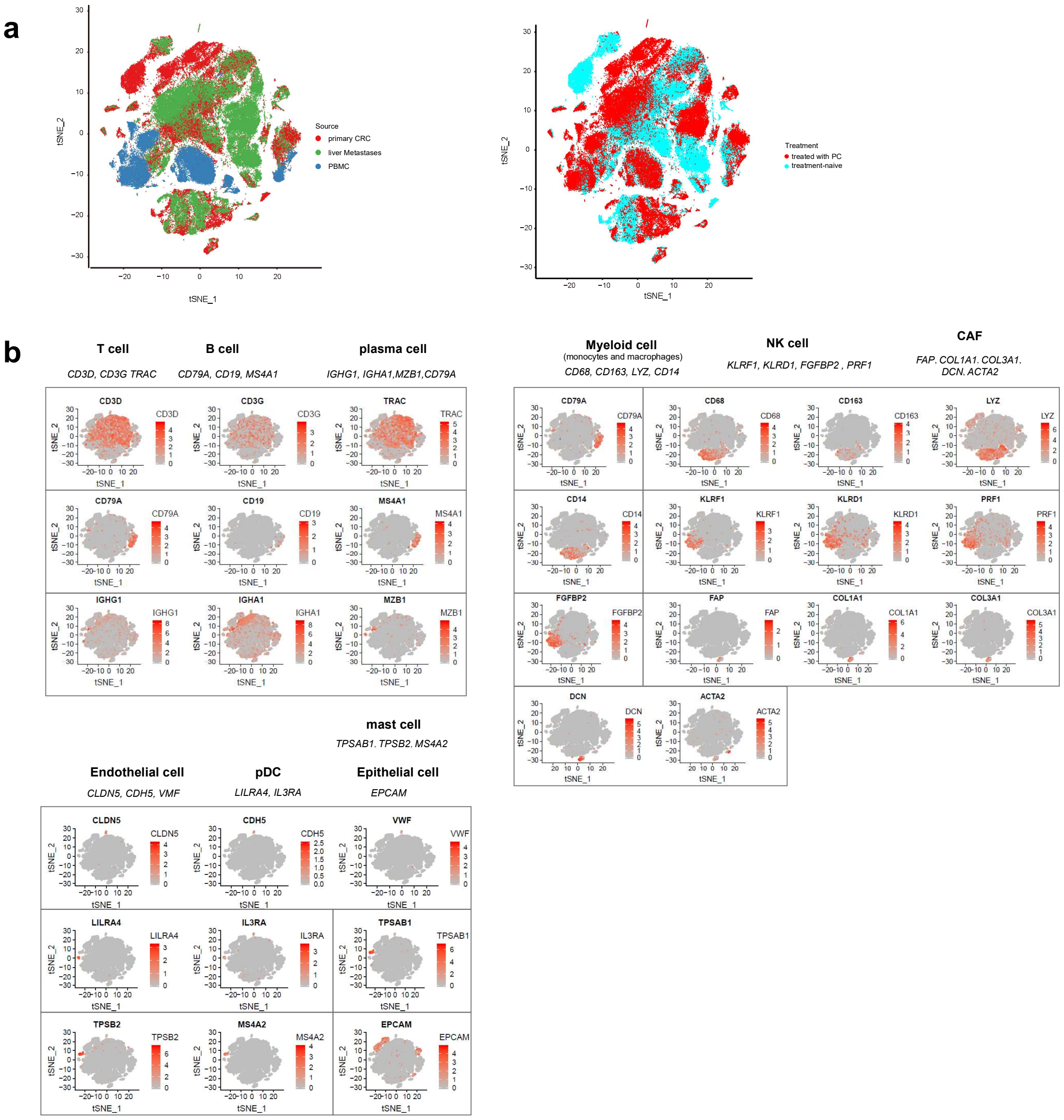

Figure S1. Overview of the single cell atlas from primary CRC, matched liver metastases and blood samples. Related to Figure 1. **a**, tSNE of cells colored by tissue, treatment status. **b**, Feature tSNE plots of selected typical canonical markers of each of the major cell type.

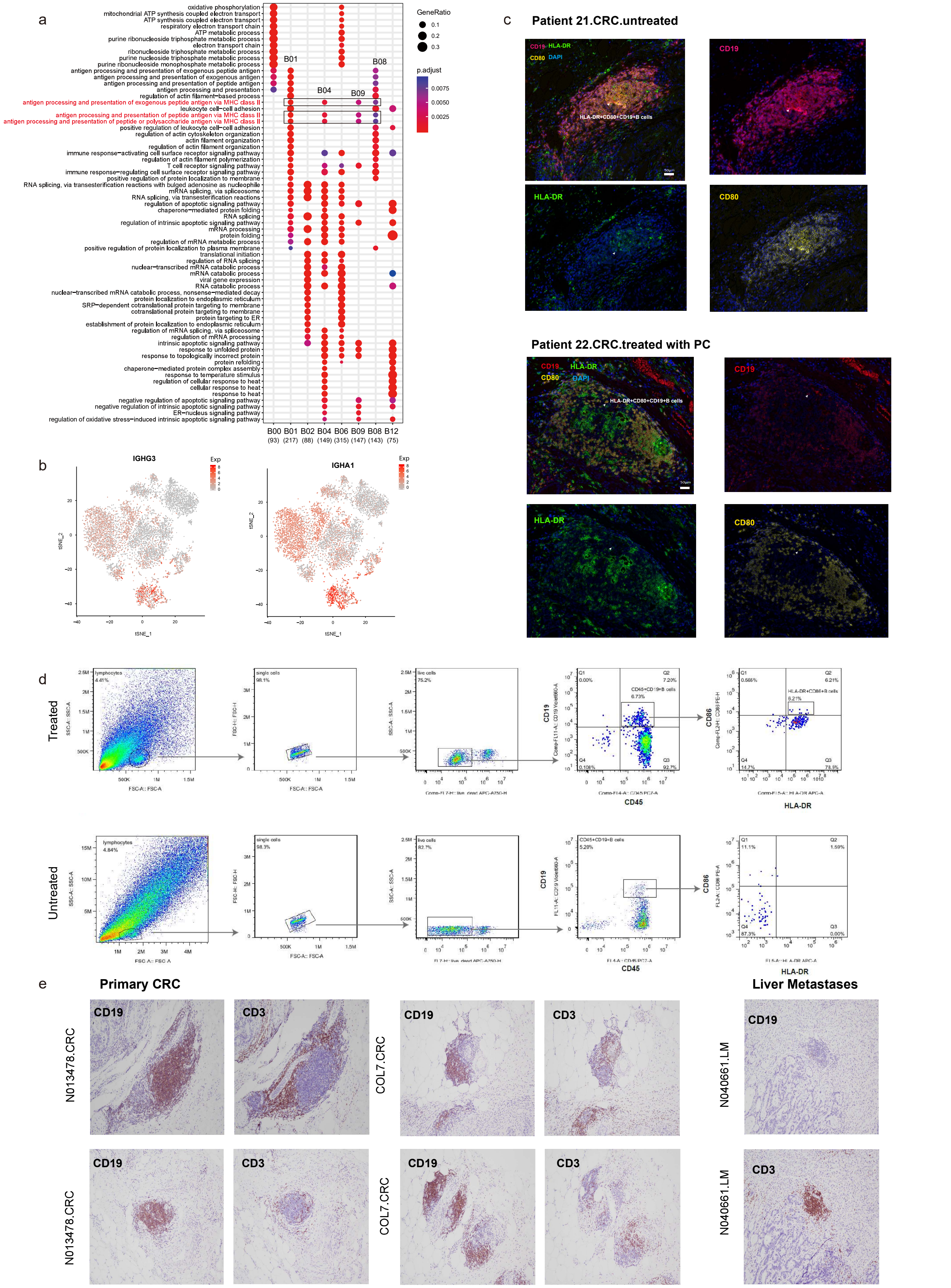

Figure S2. The generation of activated B cells after preoperative chemotherapy. Related to Figure 2. a, Gene ontology (GO) analysis of DEGs for each of the 8 B cell clusters distributed in tissue. Selected GO terms with Benjamini-Hochberg –corrected P values <0.05 (one-sided Fisher's exact test) are shown. **b**, Feature plot show the expression levels of IGHG3 and IGHA1 across 7,454 single B cells illustrated in t-SNE plots. **c**. Representative multiplex immunofluorescence assay of TLSs for the following markers: CD19 (red/magenta), CD80 (yellow), HLA-DR (green), and DAPI (blue), white arrows label a representative case of HLA-DR+CD80+CD19+B cell. In a treatment-naïve patient (top), there are only a few activated B cells (HLA-DR+CD80+CD19+B cells) in TLSs, but in the patient treated with PC (bottom), many HLA-DR+CD80+CD19+ B cells can be found in TLSs by visual inspection. **d**, Flow cytometry of tumor-infiltrating B cells with or without chemotherapy. The right shows staining for B cells gated for activation markers. **e**, The immunohistochemical (IHC) showed that CD19+ B cells were localized in tertiary lymphoid structures (TLSs) of tumors, and were colocalized with T cells. In the liver metastases, we also identify mature TLSs, but consistent with our analyses in single cell RNA-seq data, the B cells were few infiltrated in liver metastases.

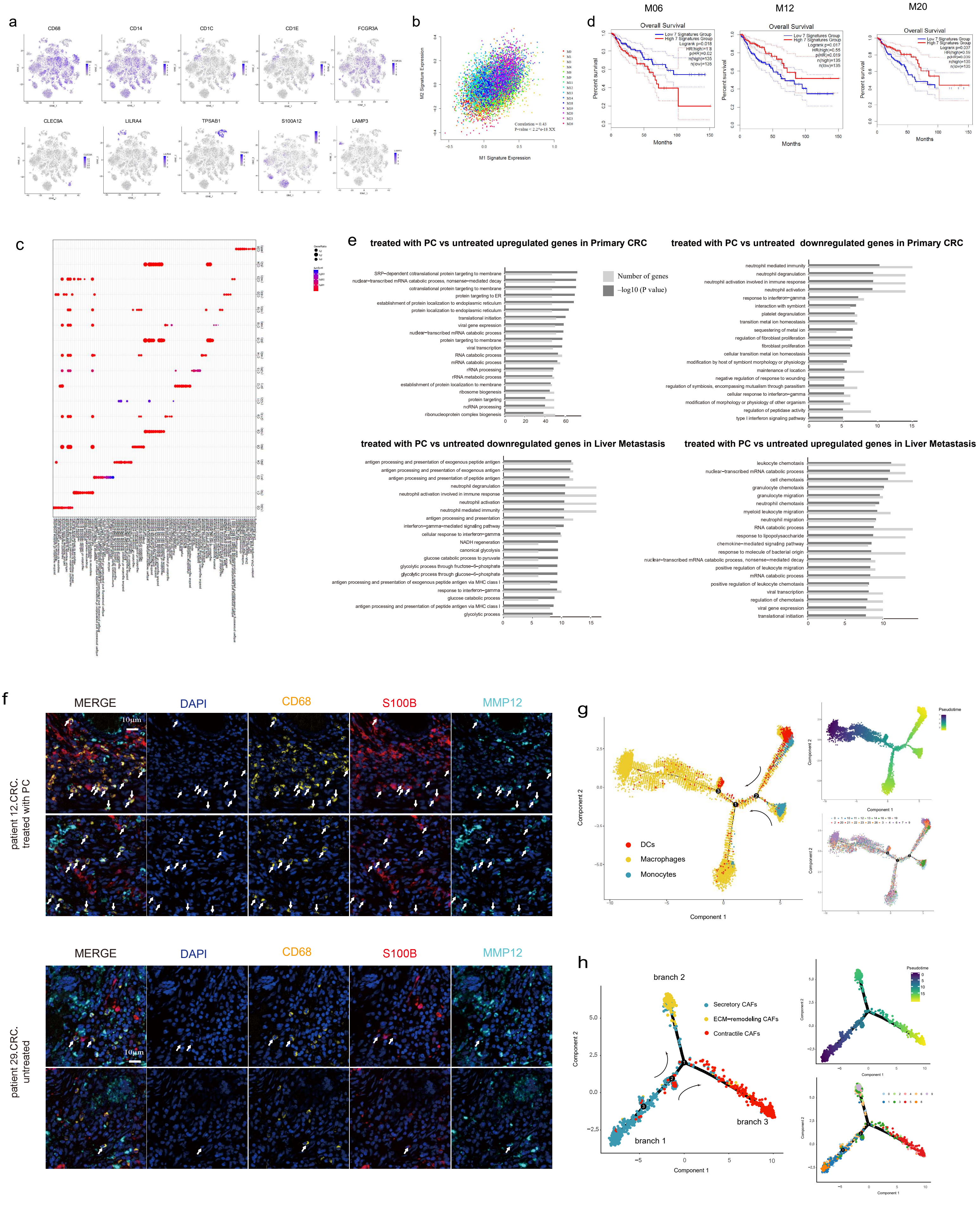

Figure S3. Compositions and functions of TAMs. Related to Figure 3 and Figure 4. a, tSNE plot of expression levels of selected genes in myeloid cells. **b**, Scatterplot of normalized mean expression of M1 and M2 signatures per cell (dot). The signature genes of M1 and M1 refer to Azizi et al., 2018. **c**, GO terms for genes in each cluster of TAMs are shown. Differentially expressed genes with P < 0.01 and log2 (fold change) < − 1 or log2 (fold change) > 1 were used. **d**, The Kaplan-Meier overall survival curves of TCGA COAD patients grouped by the gene signatures of M06, M12 and M20 respectively. The gene signatures significant separated patients into high- and low groups with different survival time with the P values = 0.05. **e**, GO analyses for genes that were differentially expressed between TAMs from tumor treated with PC versus treatment-naive tumors in the primary CRC (left) and liver Metastasis (right), respectively. Benjamini–Hochberg corrected P < 0.01. **f**, Immunofluorescence labelling of CD68, S100B, MMP12 and DAPI. Related to Figure 3g. g, The pseudotime development trajectory of myeloid cells based on the expression of the top 50 marker genes in each subpopulation. **h**, Pseudotime ordering of CAFs. Cells are colored based on cluster identities (left), pseudotime (right) and treatment status (right).

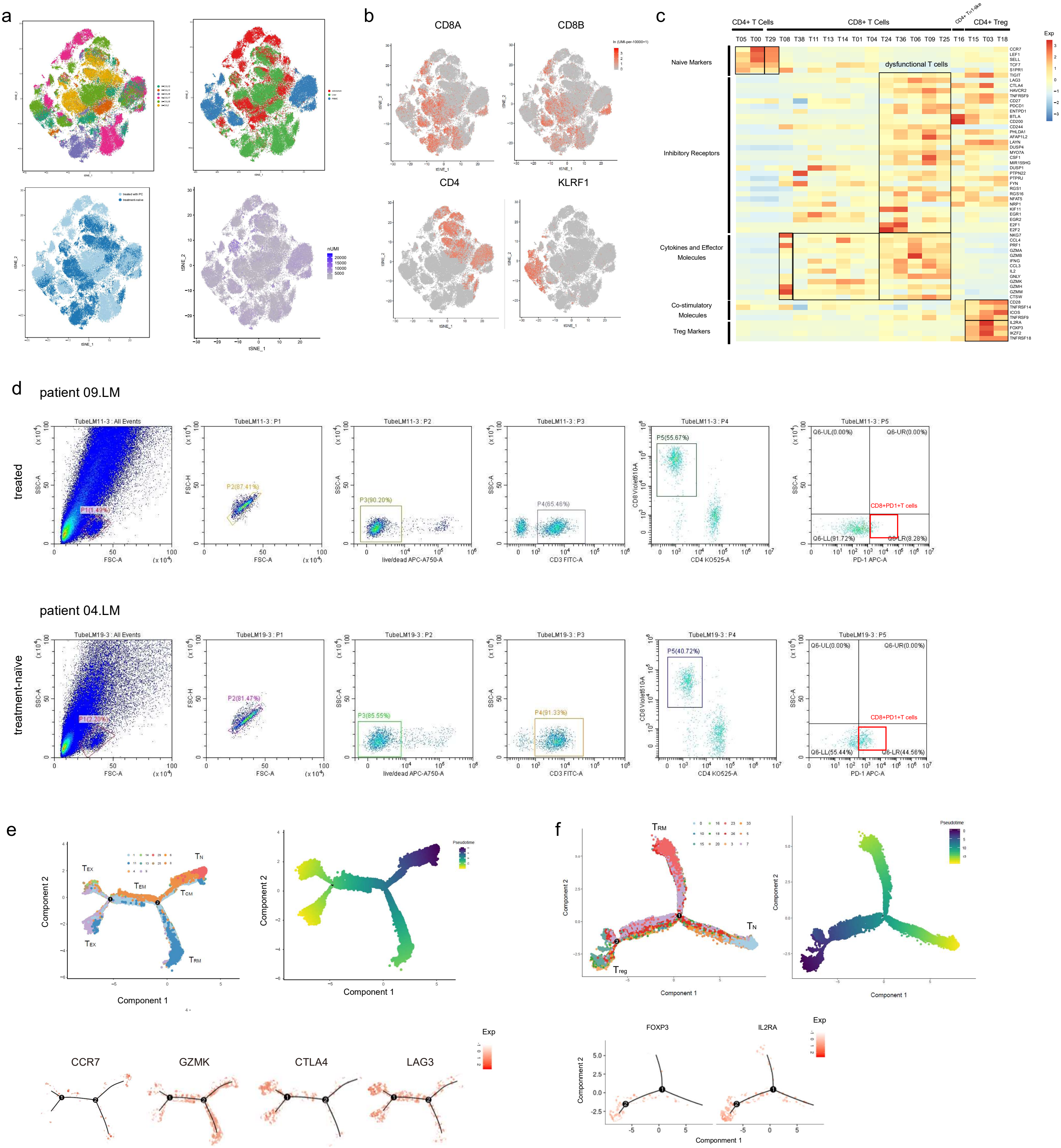

Figure S4. The annotation and differentiation trajectories of T cells. Related to Figure 5. a, tSNE of T cells colored by patient identify, tissue, treatment status, and number of UMI. **b**, Feature plots showing the expression of CD4, CD8A, CD8B and KLRF1. **c**, Heatmap showing the relative expression of T cell function-associated genes in each cell clusters. The relative expression was defined as the gene-wise (row) Z-score of normalized UMI counts (In(UMI-per-10000+1)) across T cell subtypes. Black boxes highlight the prominent patterns defining known T cell subtypes. **d**, Flow cytometry of CD8+PD1+T cells in tumors treated with and without chemotherapy. **e**,**f**, Developmental trajectory of CD8+ T cells (**e**) and CD4+ T cells (**f**) along pseudo-time in two-dimensional space. Expression of naïve markers, effector genes and markers related to dysfunctional genes on the trajectory maps. Cells are colored by the normalized UMI (unique molecular identifier) counts. Cells orders are inferred from the expression of most variable genes across all cells.

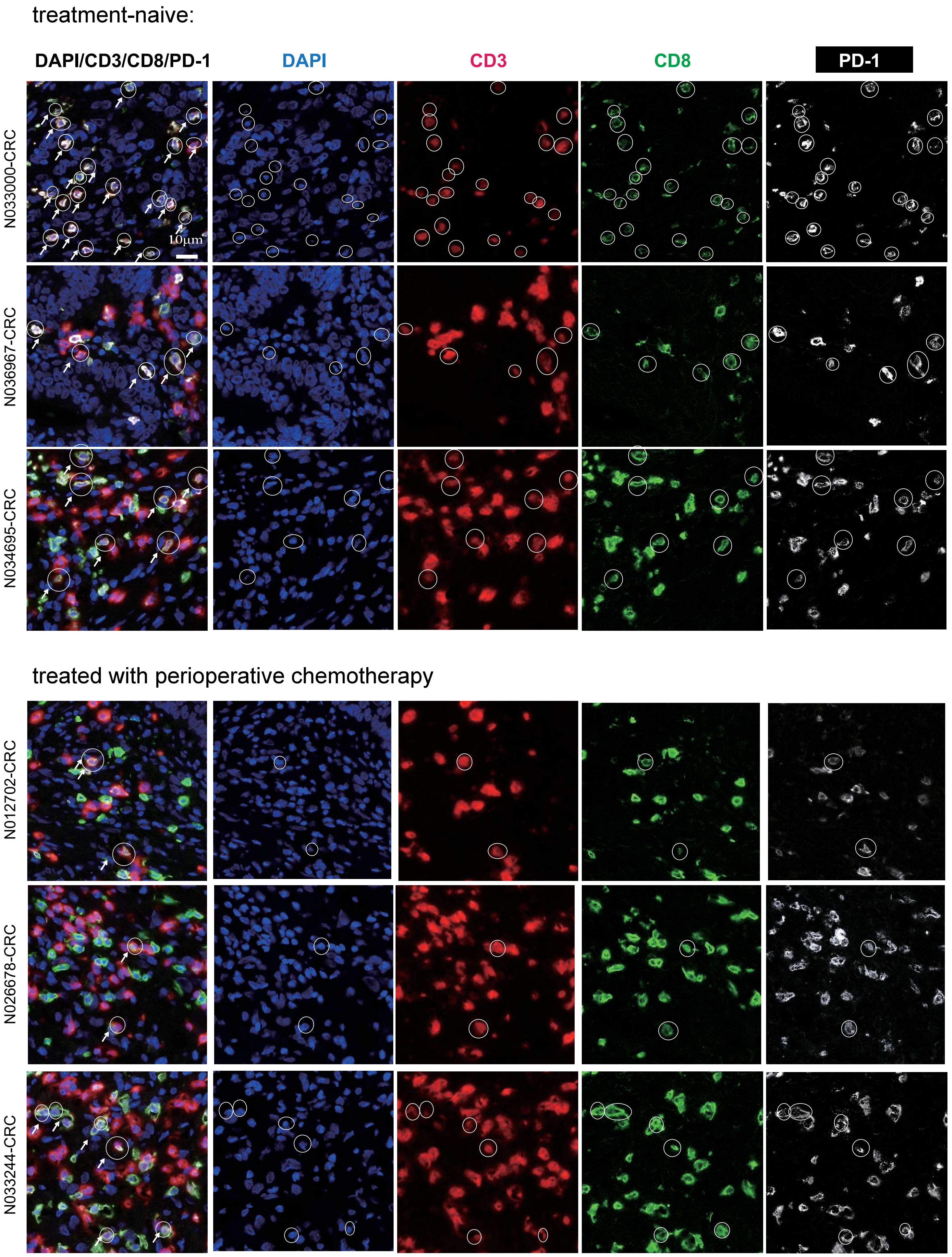

Figure S5. Immunofluorescence labelling of CD3, CD8, PD-1 and DAPI. Related to Figure 5. Nuclei are stained with DAPI. Images are maximum z-stack projections of 6 μm. Scale bar, 10 μm. (CRC: the sample from the primary CRC).

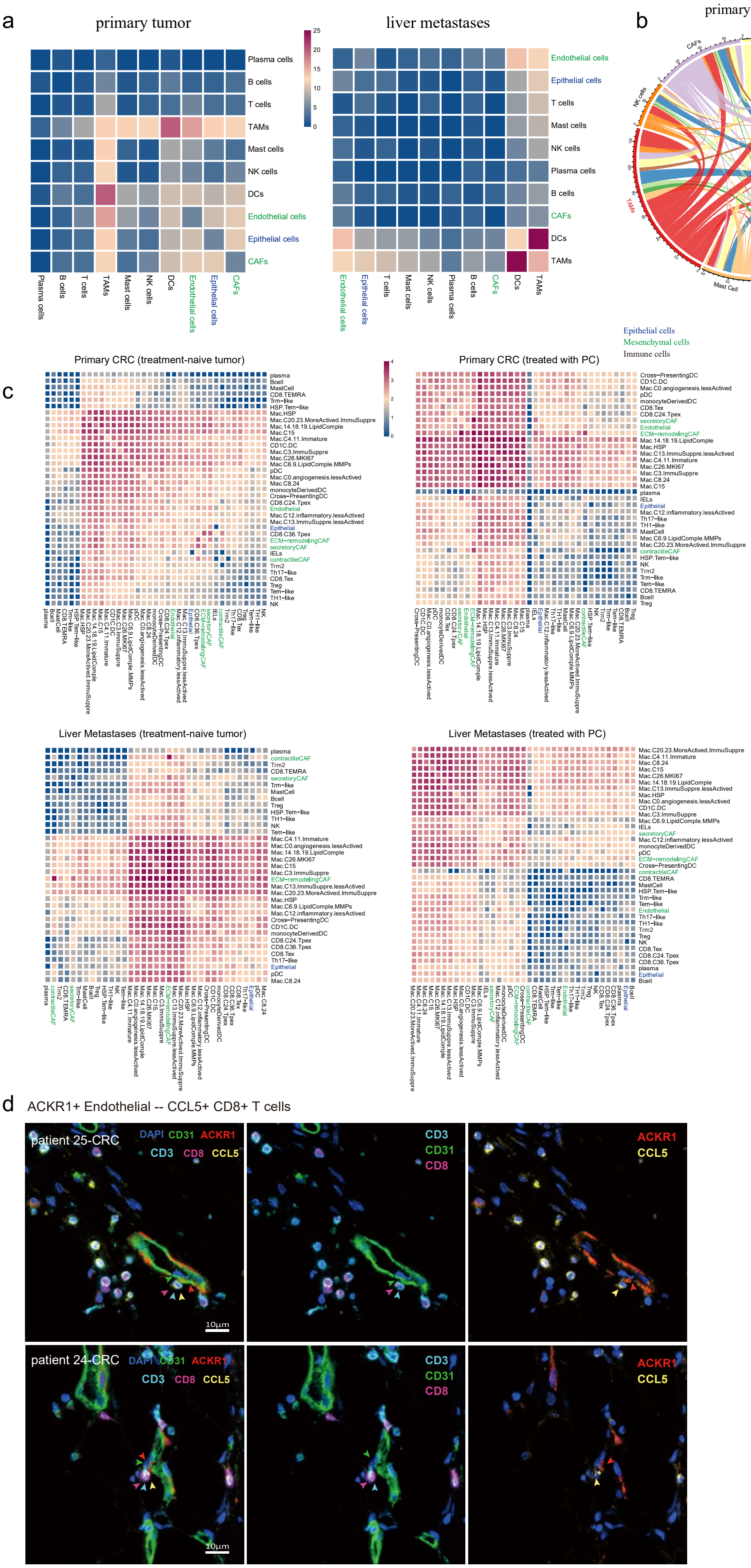

Figure S6. Cell-cell signaling networks. Related to Figure 6. a, Heatmap showing the number of cell-cell interactions within major cell types (T cells, B cells, plasma cells, NK cells, mast cells, CAFs, endothelial cells, TAMs, epithelial cells and DCs). **b**, Network visualization of cell crosstalk in primary CRC and liver metastases. Colored lines indicate predicted ligand and receptor pairs among different cell types. **c**, Heatmap showing the number of all possible cell-cell interactions within cell sub populations, based on the predication of CellphoneDB. The cell classification was based on the annotation above. **d**, Multicolor IHC staining of ACKR1 and CCL5 expression in Endothelial cells and T cells, exemplified by Patient 25 and Patient 24.
